# Double-Granule Conditional-Entropies Based on Three-Level Granular Structures

**DOI:** 10.3390/e21070657

**Published:** 2019-07-03

**Authors:** Taopin Mu, Xianyong Zhang, Zhiwen Mo

**Affiliations:** 1 School of Mathematical Sciences, Sichuan Normal University, Chengdu 610066, China; 2 Institute of Intelligent Information and Quantum Information, Sichuan Normal University, Chengdu 610066, China

**Keywords:** rough set theory, information theory, conditional entropy, uncertainty, granular computing, three-level granular structures

## Abstract

Rough set theory is an important approach for data mining, and it refers to Shannon’s information measures for uncertainty measurements. The existing local conditional-entropies have both the second-order feature and application limitation. By improvements of hierarchical granulation, this paper establishes double-granule conditional-entropies based on three-level granular structures (i.e., micro-bottom, meso-middle, macro-top), and then investigates the relevant properties. In terms of the decision table and its decision classification, double-granule conditional-entropies are proposed at micro-bottom by the dual condition-granule system. By virtue of successive granular summation integrations, they hierarchically evolve to meso-middle and macro-top, to respectively have part and complete condition-granulations. Then, the new measures acquire their number distribution, calculation algorithm, three bounds, and granulation non-monotonicity at three corresponding levels. Finally, the hierarchical constructions and achieved properties are effectively verified by decision table examples and data set experiments. Double-granule conditional-entropies carry the second-order characteristic and hierarchical granulation to deepen both the classical entropy system and local conditional-entropies, and thus they become novel uncertainty measures for information processing and knowledge reasoning.

## 1. Introduction

Rough set theory can effectively implement data mining for the imprecise, inconsistent, and incomplete information [1], and it has been extensively applied in artificial intelligence and machine learning [2,3,4,5,6,7,8]. In rough set theory, attribute reduction based on decision tables is a main topic for approximate reasoning and knowledge discovery, and there are three main construction strategies: from the positive region, information measure, and a discernibility matrix  [9,10,11,12,13,14,15]. By virtue of the discernibility matrix, Wei et al. [16] proposed an incremental reduction algorithm for dynamic data; Ma et al. [17] utilized the compressed binary discernibility matrix to construct an incremental reduction algorithm for group dynamic data; moreover, Nie and Zhou [18] proposed a new discernibility matrix defined by local conditional-entropies to compute the reduction core.

Information theory originated from Shannon’s entropy system [19], and it provides an effective method for uncertainty measurement, such as in attribute reduction. Currently, information theory has been introduced into rough set theory for uncertainty analyses and information processing [20,21,22,23,24,25]. As far as attribute reduction is concerned, Miao [26] offered the informational representation of knowledge reduction and decision reduction, where entropy and mutual-information are highlighted; Wang et al. [27] conducted a comparative study on attribute reduction from the algebra and information viewpoints, where the conditional-entropy acts as a main tool; Jiang et al. [28] presented the relative decision entropy to propose a feature selection algorithm; Slezak [29] used the conditional-entropy to define approximate reducts; moreover, Qian and Shu [30] provided the mutual information criterion to evaluate candidate features in incomplete data. In general, the entropy, conditional-entropy, and mutual-information together constitute the classical information system with integrality and comprehensiveness, and they can function on rough set applications (such as attribute reduction) but may exhibit different emphases in different application scenarios. In addition, information-theoretic measures have multiple variational forms [31,32,33,34,35]. As far as conditional-entropies are concerned, they are extensively applied in rough set theory from multiple pointcuts [26,27,29,31,34,36,37,38,39], while uncertainty measurement and reduction construction still serve as two basic issues. Aiming at probabilistic rough sets, Deng and Yao [40,41] used Shannon’s entropy and conditional-entropy to interpret and determine probabilistic thresholds by an information-theoretic approach, and Ma et al. [42] considered variants of conditional-entropies to construct heuristic reduction algorithms for the probabilistic model. In particular, local conditional-entropies are put forward by adopting double condition-granules and their union locality [18], and they can distinctively determine a new discernibility matrix for reduction core computation; moreover, the information measures exhibit a novel feature of second-order expressions, especially when compared to the traditional entropy system with only single-granule descriptions [19,26,27].

Granular computing is a structural methodology of hierarchical computing and information processing [43,44], and its technology of multi-granularity and multiple levels is useful for uncertainty analyses and knowledge acquisition regarding data. In rough set theory, the information granulation is of extensive concern [45,46,47,48,49], and the granulation monotonicity plays an important role in attribute reduction [12,50,51,52]. In particular, a decision table acts as a formal background of data mining [12,53,54,55], and it involves condition/decision granules and classifications from granular structures. According to granular computing, Zhang and Miao [56] introduced three-layer granular structures of decision tables, and they further hierarchically constructed three-way informational measures based on weighted-entropies; moreover, Wang et al. [57] utilized three-layer granular structures to research three-way weighted combination-entropies. These studies adhere to three-level analyses, and the latter are directly related to granular computing [43] and three-way decisions [58], as well as their interplay. Recently, Yao [59] discussed three-way granular computing by making use of two particular types of three granules and three levels, where thinking in three levels results in an important model. Additionally, three-level analyses were extensively utilized in the location allocation and programming/optimization modeling [60,61,62].

According to [18], the new discernibility matrix is used for reduction core calculations, and its creative implementation mainly depends on local conditional-entropies. Therefore, local conditional-entropies focus on the granule-union locality rather than their underlying double-granule interaction, and the latter more essentially adheres to the second-order characteristic; moreover, they lack the condition granulation to restrict their uncertainty measurement function and information procession prospect based on knowledge. Motivated by the two issues, this paper utilizes the two-granular essence and three-hierarchical evolution to propose double-granule conditional-entropies based on three-level granular structures. Regarding the contribution, this novel type of information measures improves local conditional-entropies from both the granular interaction and hierarchical/conditional granulation, and they will achieve multiple important properties (including the integration hierarchy, number distribution, calculation algorithm, three bounds, and granulation non-monotonicity) to offer both robust measurement functions and knowledge-application prospects. Moreover, three-level granular structures here (including micro-bottom, meso-middle, macro-top) adopt only the condition part of decision table, and thus they differ from and push forward the previous ones, which include both the condition and decision parts [56].

The remainder of this paper is organized as follows. Section 2 reviews the decision table and local conditional-entropies; Section 3 proposes and studies double-granule conditional-entropies from three-level granular structures; Section 4 provides a decision table example for mechanism illustration; Section 5 makes data experiments for effectiveness verification; finally, Section 6 concludes this paper.

## 2. Decision Table and Its Existing Entropy Measures

Rough set theory [1] focuses on the data that are represented in an information table

$$(U,AT,\left\{V_{a}:a\in AT\right\},\left\{I_{a}:a\in AT\right\});$$

*U* is the universe with finite objects, AT is the finite attribute set, $V_{a}$ is the value domain for a∈AT, and $I_{a}:U\to V_{a}$ is an information function to endow each object *x* with a value $I_{a}\left(x\right)=a\left(x\right)$ on attribute *a*. The decision table is a special type of information table with AT=C∪D and C∩D=∅, where *C* and *D* denote the sets of condition attribute and decision attribute, respectively, and it is simply denoted by (U,C∪D) in this paper. Furthermore, the granulation construction usually considers two parts.

(1) The condition attribute subset A⊆C induces an equivalence relation
  IND(A)={(x,y)∈U×U:∀a∈A,a(x)=a(y)},
  and the latter provides the condition granulation or partition $U/IND\left(A\right)=\left\{A_{i}:\;i=1,\ldots ,n\right\}$, where $A_{i}={\left[x\right]}_{A}^{i}$ represents the equivalence granule to exhibit number |U/IND(A)|=n.
(2) Similarly, the decision attribute set *D* induces the equivalence relation IND(D) and further decision classification $U/IND\left(D\right)=\left\{D_{j}:\;j=1,\ldots ,m\right\}$, which consists of |U/IND(D)|=m decision classes.

The decision table (U,C∪D) and its granulation from A⊆C and *D* constitute the basic background for information measure construction. The probability space $\left(U,2^{U},P\right)$ establishes the usual probability framework, where

(1) $$\begin{array}{c} P:2^{U}\to Q,\;P\left(X\right)=\frac{\left|X\right|}{\left|U\right|},\;\forall X\subseteq U, \end{array}$$

and thus two usual probabilities are

(2) $$\begin{array}{c} P\left(A_{i}\right)=\frac{|A_{i}|}{\left|U\right|},P\left(D_{j}/A_{i}\right)=\frac{|A_{i}\cap D_{j}|}{|A_{i}|}. \end{array}$$

**Definition** **1** ([26,27,56])**.** *The entropy on condition A, conditional-entropy on D given A, and mutual-information between A and D are respectively defined by*

(3) $$\begin{array}{c} \begin{array}{cc} H(A) & =-\sum_{i=1}^{n}P\left(A_{i}\right)\log_{2}P\left(A_{i}\right),\\ H(D/A) & =-\sum_{i=1}^{n}\left(P\left(A_{i}\right)\sum_{j=1}^{m}P\left(D_{j}/A_{i}\right)\log_{2}P\left(D_{j}/A_{i}\right)\right),\\ I(A;D) & =H(D)-H(D/A), \end{array} \end{array}$$

*where*

$$H\left(D\right)=-\sum_{j=1}^{m}P\left(D_{j}\right)\log_{2}P\left(D_{j}\right).$$

**Theorem** **1** ([26,27,56])**.** *The entropy, conditional-entropy, and mutual-information have granulation monotonicity. Concretely,*

(4) $$\begin{array}{c} U/IND(A)⪰U/IND(B)⟹H(B)\geq H(A),\;H(D/B)\leq H(D/A),\;I(B;D)\geq I(A;D). \end{array}$$

In terms of the decision table (U,C∪D), the classical system of Shannon entropies has been introduced into rough set theory, as shown by Definition 1 and Theorem 1. As three basic information measures, the entropy, conditional-entropy, and mutual-information have uncertainty semantics and granulation monotonicity, so they are extensively used in attribute reduction and heuristic algorithms [26,27,42]. The granulation relation U/IND(A)⪰U/IND(B) is equivalent to IND(A)⊇IND(B), that is,

$$\forall B_{i^{*}}\in U/IND\left(B\right),\;\exists A_{i}\in U/IND\left(A\right),\;s.t.,\;B_{i^{*}}\subseteq A_{i},$$

and it is usually induced by A⊆B⊆C; furthermore, relevant granulation monotonicity/non-monotonicity becomes an important index to assess and apply uncertainty measures.

According to the decision table and its formal structure, Zhang and Miao [56] recently introduced three-level granular structures, i.e.,

$$\mathrm{micro-bottom}\;(A_{i},D_{j}),\;\mathrm{meso-middle}\;(U/IND\left(A\right),D_{j}),\;\mathrm{macro-top}\;(U/IND(A),U/IND(D)),$$

and further investigated weighted-entropy constructions. As a result, the previous entropy system (Equation (3)) is actually located at macro-top and has an equivalent construction from the weighted-entropy system; at meso-middle, Zhang et al. [10] established three-way informational class-specific reducts to be compared with the algebraic class-specific reducts [9].

In particular, Nie and Zhou [18] proposed a new discernibility matrix for computing the reduction core, and they tactfully utilized a kind of novel information of so-called local conditional-entropy. As our preliminary, the relevant entropy and matrix are reviewed as follows, where let $U/IND\left(C\right)\;=\;\left\{C_{k}:\;k=1,..,r\right\}$ and the cardinality form is mainly adopted.

**Definition** **2** ([18])**.** *The local conditional-entropy on decision table (U,C∪D) is defined by:*

(5) $$\begin{array}{c} \begin{array}{cc} \forall C_{p},C_{q}\in U/IND\left(C\right) & \;(1\leq p,q\leq r),\\ H_{C_{p}\cup C_{q}}\left(D/C\right)= & -\frac{|C_{p}|}{|C_{p}\cup C_{q}|}\sum_{j=1}^{m}\frac{|C_{p}\cap D_{j}|}{|C_{p}|}\log_{2}\frac{|C_{p}\cap D_{j}|}{|C_{p}|}\\ & -\frac{|C_{q}|}{|C_{p}\cup C_{q}|}\sum_{j=1}^{m}\frac{|C_{q}\cap D_{j}|}{|C_{q}|}\log_{2}\frac{|C_{q}\cap D_{j}|}{|C_{q}|}. \end{array} \end{array}$$

**Definition** **3** ([18])**.** *The discernibility matrix $DM={\left(r_{i^{\prime }j^{\prime }}\right)}_{\left|U|\times |U\right|}$ on decision table (U,C∪D) is defined by:*

(6) $$r_{i^{\prime }j^{\prime }}=\left\{\begin{array}{cc} c\in C, & if\;min(|dx_{i^{\prime }}|,|dx_{j^{\prime }}\left|\right)=1,c\left(x_{i^{\prime }}\right)\neq c\left(x_{j^{\prime }}\right),D\left(x_{i^{\prime }}\right)\neq D\left(x_{j^{\prime }}\right),\\ c\in C, & if\;min(|dx_{i^{\prime }}|,|dx_{j^{\prime }}\left|\right)>1,c\left(x_{i^{\prime }}\right)\neq c\left(x_{j^{\prime }}\right),D\left(x_{i^{\prime }}\right)\neq D\left(x_{j^{\prime }}\right),\\ & H_{{\left[x_{i^{\prime }}\right]}_{C}\cup {\left[x_{j^{\prime }}\right]}_{C}}\left(D/\left(C-\left\{c\right\}\right)\right)>H_{{\left[x_{i^{\prime }}\right]}_{C}\cup {\left[x_{j^{\prime }}\right]}_{C}}\left(D/C\right),\\ \emptyset ,\;\;\;\; & otherwise \end{array}\right.$$

*where $dx=\{D\left(y\right):y\in {\left[x\right]}_{C}\}$ (∀x,y∈U) represents the set of decision values induced by conditional class ${\left[x\right]}_{C}$ while |dx| means the corresponding cardinality [63]. In Equation (6), let ${\left[x_{i^{\prime }}\right]}_{C}=C_{p}$, ${\left[x_{j^{\prime }}\right]}_{C}=C_{q}$, and then*

(7) $$H_{{\left[x_{i^{\prime }}\right]}_{C}\cup {\left[x_{j^{\prime }}\right]}_{C}}\left(D/\left(C-\left\{c\right\}\right)\right)=H_{C_{p}\cup C_{q}}\left(D/\left(C-\left\{c\right\}\right)\right)=-\sum_{j=1}^{m}\frac{|\left(C_{p}\cup C_{q}\right)\cap D_{j}|}{|C_{p}\cup C_{q}|}\log_{2}\frac{|\left(C_{p}\cup C_{q}\right)\cap D_{j}|}{|C_{p}\cup C_{q}|}$$

*is determined to represent the conditional-entropy of local decision table when accompanied by new universe $C_{p}\cup C_{q}$ after deleting attribute c; moreover, $H_{{\left[x_{i^{\prime }}\right]}_{C}\cup {\left[x_{j^{\prime }}\right]}_{C}}\left(D/C\right)=H_{C_{p}\cup C_{q}}\left(D/C\right)$ is clear according to Equation (5).*

## 3. Double-Granule Conditional-Entropies Based on Three-Level Granular Structures

The local conditional-entropy in Equation (5) implements effective uncertainty descriptions to guide the in-depth discernibility matrix and core calculation [18], thus exhibiting fundamental significance. However, this basic measure has three flawed aspects, and corresponding improvements for general applications.

(1) According to Equation (5), the locality mainly refers to less range $C_{p}\cup C_{q}$ in universe U. More essentially, we can stand on the dual granules $C_{p}$ and $C_{q}$ to propose a novel notion of double-granule conditional-entropies, and it differs from the usual entropy system with only the single-granule representation which implies a kind of first-order style. Moreover, the measure properties are lacking in [18], and we will provide in-depth properties such as restriction bounds and granulation non-monotonicity.
(2) Regarding granular structures, all decision classes $D_{j}$ (j=1,⋯,m) (or decision classification U/IND(D)) are considered, but condition granules involve only two factors $C_{p}$ and $C_{q}$. A condition partition U/IND(C)) needs considering in practice to provide a system description of knowledge granulation, so we also focus on granulation U/IND(C) to introduce three-level granular structures for hierarchical constructions of double-granule conditional-entropies.
(3) Finally, the initial concept is limited to only C for expressing the discernibility matrix and reduction core, and a general subset A⊆C has better theoretical and practical prospects, especially for the knowledge-based applications (such as attribute reduction or feature selection).

Along the above thoughts, this section mainly establishes double-granule conditional-entropies based on a universal attribute-subset A⊆C and investigates relevant algorithms and properties, and we particularly use a kind of three-level granular structures.

**Figure 1:**
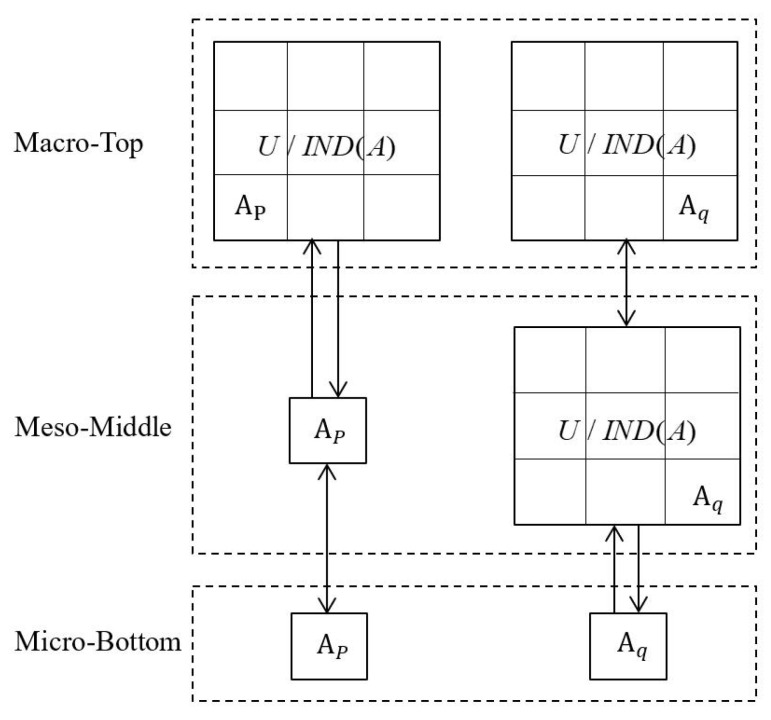
Schematic diagram of three-level granular structures.

From a viewpoint of only condition granulation, basic descriptions of three-level granular structures are provided in Table 1, and relevant concepts are usually intuitionistic and descriptive according to a supporting figure with granular structures: Figure 1. Micro-bottom $\left(A_{p},A_{q}\right)$ focuses on only two granules, meso-middle

$$\left(A_{p},U/IND\left(A\right)=\left\{A_{q}:q=1,\cdots ,n\right\}\right)$$

consists of one granule and a partition, while macro-top

$$\left(U/IND\left(A\right)=\left\{A_{p}:p=1,\cdots ,n\right\},U/IND\left(A\right)=\left\{A_{q}:q=1,\cdots ,n\right\}\right)$$

considers the same partition with different construction origins. The three-level granular structures carry a kind of hierarchical integration (or decomposition) relationship, and they provide n×n, *n*, and one parallel patterns, respectively; they will be presented in a table form with the n×n mainbody data as well as the edge statistics. Moreover, they differ from the existing three-level granular structures for decision tables, which consider not only the condition granulation (with $A_{i}$ and U/IND(A)) but also decision granulation (with $D_{j}$ and U/IND(D)) [56].

### 3.1. Double-Granule Conditional-Entropy at Micro-Bottom

The local conditional-entropies are actually at only micro-bottom, i.e., $\left(C_{p},C_{q}\right)$ regarding C. As a basis of hierarchical development, this subsection improves local conditional-entropies to construct double-granule conditional-entropies at micro-bottom $\left(A_{p},A_{q}\right)$ (p,q∈{1,⋯,n}), which comes from an arbitrary condition-attribute subset A⊆C. We first suppose weight coefficients

(8) $$\begin{array}{c} \begin{array}{c} \omega_{p}=\frac{|A_{p}|}{|A_{p}\left|+\right|A_{q}|},\omega_{q}=\frac{|A_{q}|}{|A_{p}\left|+\right|A_{q}|}, \end{array} \end{array}$$

where

$$\omega_{p}+\omega_{q}=1.$$

**Definition** **4.** *At micro-bottom $\left(A_{p},A_{q}\right)$, the double-granule conditional-entropy is defined by*

(9) $$\begin{array}{cc} H_{\left(A_{p},A_{q}\right)}\left(D/A\right) & =-\omega_{p}\sum_{j=1}^{m}P\left(D_{j}/A_{p}\right)\log_{2}P\left(D_{j}/A_{p}\right)-\omega_{q}\sum_{j=1}^{m}P\left(D_{j}/A_{q}\right)\log_{2}P\left(D_{j}/A_{q}\right)\\ & =-\frac{|A_{p}|}{|A_{p}\left|+\right|A_{q}|}\sum_{j=1}^{m}\frac{|A_{p}\cap D_{j}|}{|A_{p}|}\log_{2}\frac{|A_{p}\cap D_{j}|}{|A_{p}|}-\frac{|A_{q}|}{|A_{p}\left|+\right|A_{q}|}\sum_{j=1}^{m}\frac{|A_{q}\cap D_{j}|}{|A_{q}|}\log_{2}\frac{|A_{q}\cap D_{j}|}{|A_{q}|}. \end{array}$$

**Proposition** **1.** *The double-granule conditional-entropy based on $A_{p}$ becomes*

(10) $$\begin{array}{c} \begin{array}{cc} H_{\left(A_{p},A_{p}\right)}\left(D/A\right) & =-\sum_{j=1}^{m}P\left(D_{j}/A_{p}\right)\log_{2}P\left(D_{j}/A_{p}\right)\\ & =-\sum_{j=1}^{m}\frac{|A_{p}\cap D_{j}|}{|A_{p}|}\log_{2}\frac{|A_{p}\cap D_{j}|}{|A_{p}|}. \end{array} \end{array}$$

By using probabilistic and cardinal forms, Definition 4 proposes the double-granule conditional-entropy at micro-bottom. In contrast to the local conditional-entropy in [18], our measure generally adopts the same essence but a different viewpoint. In other words, Equation (9) with forms $\left(A_{p},A_{p}\right)$ and $|A_{q}\left|+\right|A_{p}|$ is equivalent to Equation (5) with styles $A_{p}\cup A_{p}$ and $|A_{q}\cup A_{p}|$ when

$$A_{q}\neq A_{p}⟹|A_{q}\left|+\right|A_{p}\left|=\right|A_{q}\cup A_{p}|,$$

but the former becomes different and coherent when

$$A_{q}=A_{p}⟹|A_{q}\left|+\right|A_{p}\left|=2\right|A_{q}\cup A_{p}\left|>\right|A_{q}\cup A_{p}|;$$

moreover, it more tends to the double-granule description rather than the granule-union locality. In Equation (9), conditional-information measures

$$-\sum_{j=1}^{m}P\left(D_{j}/A_{p}\right)\log_{2}P\left(D_{j}/A_{p}\right),-\sum_{j=1}^{m}P\left(D_{j}/A_{q}\right)\log_{2}P\left(D_{j}/A_{q}\right)$$

represent the uncertainty of decision classification U/IND(D) regarding condition granules $A_{p}$ and $A_{q}$, respectively, and they are integrated into $H_{\left(A_{p},A_{q}\right)}\left(D/A\right)$ by two complementary weight coefficients $\omega_{p}$ and $\omega_{q}$. As a result, $H_{\left(A_{p},A_{q}\right)}\left(D/A\right)$ embodies a kind of information fusion of double-granule $A_{p}$, $A_{q}$ to describe decision classification U/IND(D) and its uncertainty, from the perspective of conditional information. Therefore, $H_{\left(A_{p},A_{q}\right)}\left(D/A\right)$ is naturally called the double-granule conditional-entropy, and it is actually located at micro-bottom $\left(A_{p},A_{q}\right)$. In particular, the double-granule measures utilize the double-granule fusion to capture a new feature of second-order, because main entropy systems (such as those in Equation (3)) utilize only the single-granule description which correspondingly refers to the so-called first-order information. Proposition 1 focuses on a specific case of $A_{q}=A_{p}$, and the concrete result $H_{\left(A_{p},A_{p}\right)}\left(D/A\right)$ degenerates into a one-order measure regarding conditional-entropy.

**Proposition** **2.** *At micro-bottom, double-granule conditional-entropies offer n×n values, i.e.,*

$$H_{\left(A_{p},A_{q}\right)}\left(D/A\right)\;\left(p,q=1,\cdots ,n\right).$$

Since both $A_{p}$ and $A_{q}$ have *n* granules based on p=1,⋯,n and q=1,⋯,n, $H_{\left(A_{p},A_{q}\right)}\left(D/A\right)$ offers number n×n (Proposition 2) to correspond to n×n micro-bottoms. The n×n kinds of double-granule conditional-entropies are arranged in Table 2, and the mainbody refers to an n×n square symmetric matrix where

$$H_{\left(A_{p},A_{q}\right)}\left(D/A\right)=H_{\left(A_{q},A_{p}\right)}\left(D/A\right).$$

Based on Equation (9), Algorithm 1 resorts to a “for” loop to effectively offer a double-granule conditional-entropy $H_{\left(A_{p},A_{q}\right)}\left(D/A\right)$ for two arbitrary granules $A_{p},A_{q}\in U/IND\left(A\right)$. Furthermore, we can achieve all n×n entropies values by adding two “for” loops regarding p=1,⋯,n and q=1,⋯,n.

**Algorithm 1:** Calculation of double-granule conditional-entropy at micro-bottom
**Input:** Decision table (U,C∪D), target subset A⊆C, and two special indexes   p,q∈{1,⋯,n}; **Output:** Double-granule conditional-entropy $H_{\left(A_{p},A_{q}\right)}\left(D/A\right)$ at micro-bottom $\left(A_{p},A_{q}\right)$.
1:Compute U/IND(A) to obtain two concrete granules $A_{p},A_{q}\in U/IND\left(A\right)$, and determine $\omega_{p},\omega_{q}$.2:Compute U/IND(D) to obtain all decision classes $D_{j}$ (j=1,⋯,m).3:Let $H_{p}=0$, $H_{q}=0$.4:**for**j∈{1,..,m}**do**5:  $H_{p}\leftarrow H_{p}-P\left(D_{j}/A_{p}\right)\log_{2}P\left(D_{j}/A_{p}\right)$,  $H_{q}\leftarrow H_{q}-P\left(D_{j}/A_{q}\right)\log_{2}P\left(D_{j}/A_{q}\right)$.6:**end for**7:Obtain $H_{\left(A_{p},A_{q}\right)}\left(D/A\right)=\omega_{p}H_{p}+\omega_{q}H_{q}$.8:**return**$H_{\left(A_{p},A_{q}\right)}\left(D/A\right)$.

**Theorem** **2.** *At micro-bottom, the double-granule conditional-entropy has lower and upper bounds. Concretely,*

$${\underline{H}}_{\left(A_{p},A_{q}\right)}\left(D/A\right)\leq H_{\left(A_{p},A_{q}\right)}\left(D/A\right)\leq {\bar{H}}_{\left(A_{p},A_{q}\right)}\left(D/A\right),$$

*where*

(11) $$\begin{array}{c} \begin{array}{cc} {\underline{H}}_{\left(A_{p},A_{q}\right)}\left(D/A\right) & =-\frac{|A_{p}|}{2|U|}\sum_{j=1}^{m}P\left(D_{j}/A_{p}\right)\log_{2}P\left(D_{j}/A_{p}\right)-\frac{|A_{q}|}{2|U|}\sum_{j=1}^{m}P\left(D_{j}/A_{q}\right)\log_{2}P\left(D_{j}/A_{q}\right),\\ {\bar{H}}_{\left(A_{p},A_{q}\right)}\left(D/A\right) & =-\sum_{j=1}^{m}P\left(D_{j}/A_{p}\right)\log_{2}P\left(D_{j}/A_{p}\right)-\sum_{j=1}^{m}P\left(D_{j}/A_{q}\right)\log_{2}P\left(D_{j}/A_{q}\right). \end{array} \end{array}$$

**Proof.**  $|A_{p}\left|,\right|A_{q}\left|\in [1,|U|\right)$ implies

(12) $$\begin{array}{c} \begin{array}{c} \omega_{p}=\frac{|A_{p}|}{|A_{p}\left|+\right|A_{q}|}\in \left[\frac{|A_{p}|}{2|U|},1\right),\\ \omega_{q}=\frac{|A_{q}|}{|A_{p}\left|+\right|A_{q}|}\in \left[\frac{|A_{q}|}{2|U|},1\right), \end{array} \end{array}$$

so $H_{\left(A_{p},A_{q}\right)}\left(D/A\right)\in \left[{\underline{H}}_{\left(A_{p},A_{q}\right)}\left(D/A\right),{\bar{H}}_{\left(A_{p},A_{q}\right)}\left(D/A\right)\right]$. □

In Theorem 2, the double bounds of $H_{\left(A_{p},A_{q}\right)}\left(D/A\right)$ are acquired by the enlarging and reducing of weight coefficients. Regarding Equation (12),

(13) $$\begin{array}{c} \begin{array}{c} \left(A_{q}\neq A_{p}\right)⋁\left(A_{q}=A_{p}\wedge |A_{q}\left|=\right|A_{p}|\leq \frac{\left|U\right|}{2}\right)⟹\omega_{p}\geq \frac{|A_{p}|}{\left|U\right|}>\frac{|A_{p}|}{2|U|},\omega_{q}\geq \frac{|A_{q}|}{\left|U\right|}>\frac{|A_{q}|}{2|U|}; \end{array} \end{array}$$

on the other hand,

(14) $$\begin{array}{c} \begin{array}{c} \left(A_{q}=A_{p}\right)⋀\left(|A_{q}\left|=\right|A_{p}|>\frac{\left|U\right|}{2}\right)⟹\omega_{p}=\frac{1}{2}\in \left[\frac{|A_{p}|}{2|U|},\frac{|A_{p}|}{\left|U\right|}\right),\omega_{q}=\frac{1}{2}\in \left[\frac{|A_{q}|}{2|U|},\frac{|A_{q}|}{\left|U\right|}\right). \end{array} \end{array}$$

In other words, $\omega_{p}$ and $\omega_{q}$ have theoretical lower bounds $\frac{|A_{p}|}{2|U|}$ and $\frac{|A_{q}|}{2|U|}$, respectively, but they usually have closer lower bounds $\frac{|A_{p}|}{\left|U\right|}$ and $\frac{|A_{q}|}{\left|U\right|}$, respectively. Therefore, $H_{\left(A_{p},A_{q}\right)}\left(D/A\right)$ can theoretically achieve ${\underline{H}}_{\left(A_{p},A_{q}\right)}\left(D/A\right)$, such as in the case

$$A_{p}=A_{q}=U⟹\left(\omega_{p}=\frac{1}{2}=\frac{|A_{p}|}{2|U|}\right)⋀\left(\omega_{q}=\frac{1}{2}=\frac{|A_{q}|}{2|U|}\right);$$

usually, it may be practically restricted by a better measure:

(15) $$\begin{array}{c} {\underline{H}}_{\left(A_{p},A_{q}\right)}^{\prime }\left(D/A\right)=-\frac{|A_{p}|}{\left|U\right|}\sum_{j=1}^{m}P\left(D_{j}/A_{p}\right)\log_{2}P\left(D_{j}/A_{p}\right)-\frac{|A_{q}|}{\left|U\right|}\sum_{j=1}^{m}P\left(D_{j}/A_{q}\right)\log_{2}P\left(D_{j}/A_{q}\right), \end{array}$$

which offers

(16) $$\begin{array}{c} {\underline{H}}_{\left(A_{p},A_{q}\right)}^{\prime }\left(D/A\right)\geq {\underline{H}}_{\left(A_{p},A_{q}\right)}\left(D/A\right). \end{array}$$

We below provide another upper bound of $H_{\left(A_{p},A_{q}\right)}\left(D/A\right)$, which may be better than ${\bar{H}}_{\left(A_{p},A_{q}\right)}\left(D/A\right)$ in some cases.

**Theorem** **3.** *At micro-bottom, the double-granule conditional-entropy has an upper bound. Concretely,*

(17) $$\begin{array}{c} \begin{array}{cc} H_{\left(A_{p},A_{q}\right)}\left(D/A\right) & \leq H_{\left(A_{p},A_{q}\right)}^{*}\left(D/A\right)\\ & =-\sum_{j=1}^{m}\frac{|A_{p}\cap D_{j}\left|+\right|A_{q}\cap D_{j}|}{|A_{p}\left|+\right|A_{q}|}\log_{2}\frac{|A_{p}\cap D_{j}\left|+\right|A_{q}\cap D_{j}|}{|A_{p}\left|+\right|A_{q}|}. \end{array} \end{array}$$

**Figure 2:**
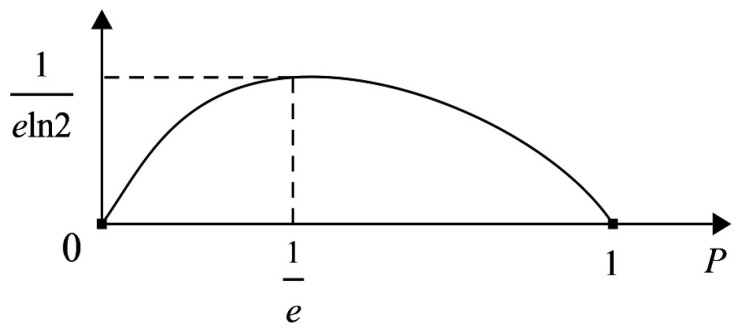
Convex figure of information function $f\left(P\right)=-P\log_{2}P$.

**Proof.**  As shown in Figure 2, function $f\left(P\right)=-P\log_{2}P$ (P∈[0,1]) is convex, where $f^{ȃ}\left(P\right)\;=\;-\;\frac{1}{P\ln2}\;<\;0$. Thus, let

$$P_{p}=P\left(D_{j}/A_{p}\right)=\frac{|A_{p}\cap D_{j}|}{|A_{p}|},P_{q}=P\left(D_{j}/A_{q}\right)=\frac{|A_{q}\cap D_{j}|}{|A_{q}|},$$

and then the famous “Jensen’s inequality” in mathematics could induce

$$\begin{array}{c} \omega_{p}+\omega_{q}=1⟹-\omega_{p}P_{p}\log_{2}P_{p}-\omega_{q}P_{q}\log_{2}P_{q}\leq -\left(\omega_{p}P_{q}+\omega_{p}P_{q}\right)\log_{2}\left(\omega_{p}P_{q}+\omega_{p}P_{q}\right), \end{array}$$

where

$$\omega_{p}P_{q}+\omega_{p}P_{q}=\frac{|A_{p}|}{|A_{p}\left|+\right|A_{q}|}\frac{|A_{p}\cap D_{j}|}{|A_{p}|}+\frac{|A_{q}|}{|A_{p}\left|+\right|A_{q}|}\frac{|A_{q}\cap D_{j}|}{|A_{q}|}=\frac{|A_{p}\cap D_{j}\left|+\right|A_{q}\cap D_{j}|}{|A_{p}\left|+\right|A_{q}|}.$$

In other words, we can get

$$\begin{array}{c} \begin{array}{cc} H_{\left(A_{p},A_{q}\right)}\left(D/A\right) & =\sum_{j=1}^{m}\left[-\omega_{p}P_{p}\log_{2}P_{p}-\omega_{q}P_{q}\log_{2}P_{q}\right]\\ & \leq \sum_{j=1}^{m}-\left(\omega_{p}P_{q}+\omega_{p}P_{q}\right)\log_{2}\left(\omega_{p}P_{q}+\omega_{p}P_{q}\right)\\ & =-\sum_{j=1}^{m}\frac{|A_{p}\cap D_{j}\left|+\right|A_{q}\cap D_{j}|}{|A_{p}\left|+\right|A_{q}|}\log_{2}\frac{|A_{p}\cap D_{j}\left|+\right|A_{q}\cap D_{j}|}{|A_{p}\left|+\right|A_{q}|}\\ & =H_{\left(A_{p},A_{q}\right)}^{*}\left(D/A\right). \end{array} \end{array}$$

□

In Theorem 3, the convex property of information function $f\left(P\right)=-P\log_{2}P$ is utilized to provide a new upper bound $H_{\left(A_{p},A_{q}\right)}^{*}\left(D/A\right)$ of central measure $H_{\left(A_{p},A_{q}\right)}\left(D/A\right)$. When comparing Equations (7) and (17), we can surprisingly discover that $H_{\left(A_{p},A_{q}\right)}^{*}\left(D/A\right)$ highly adheres to

(18) $$\begin{array}{c} \begin{array}{c} H_{A_{p}\cup A_{q}}\left(D/\left(A-\left\{a\right\}\right)\right)=-\sum_{j=1}^{m}\frac{|\left(A_{p}\cup A_{q}\right)\cap D_{j}|}{|A_{p}\cup A_{q}|}\log_{2}\frac{|\left(A_{p}\cup A_{q}\right)\cap D_{j}|}{|A_{p}\cup A_{q}|}, \end{array} \end{array}$$

which naturally comes from $H_{C_{p}\cup C_{q}}\left(D/\left(C-\left\{c\right\}\right)\right)$ (Equation (7)). In fact,

(19) $$\begin{array}{c} \begin{array}{c} H_{\left(A_{p},A_{q}\right)}^{*}\left(D/A\right)=H_{A_{p}\cup A_{q}}\left(D/\left(A-\left\{a\right\}\right)\right) \end{array} \end{array}$$

when $A_{p}\neq A_{q}$; when

$$A_{q}=A_{p}⟹\frac{|A_{p}\cap D_{j}\left|+\right|A_{q}\cap D_{j}|}{|A_{p}\left|+\right|A_{q}|}=\frac{|A_{p}\cap D_{j}|}{|A_{p}|}\geq \frac{|A_{p}\cap D_{j}|}{2|A_{p}|}=\frac{|\left(A_{p}\cup A_{q}\right)\cap D_{j}|}{|A_{p}\left|+\right|A_{q}|},$$

where $A_{p}\cup A_{q}=A_{p}$, there is a difference between two measures, and we obtain

(20) $$\begin{array}{c} H_{\left(A_{p},A_{p}\right)}^{*}\left(D/A\right)=-\sum_{j=1}^{m}\frac{|A_{p}\cap D_{j}|}{|A_{p}|}\log_{2}\frac{|A_{p}\cap D_{j}|}{|A_{p}|}\neq -\sum_{j=1}^{m}\frac{|A_{p}\cap D_{j}|}{2|A_{p}|}\log_{2}\frac{|A_{p}\cap D_{j}|}{2|A_{p}|}=H_{A_{p}\cup A_{q}}\left(D/\left(A-\left\{a\right\}\right)\right). \end{array}$$

Thus far, $H_{\left(A_{p},A_{q}\right)}\left(D/A\right)$ has one lower bound ${\underline{H}}_{\left(A_{p},A_{q}\right)}\left(D/A\right)$ and two upper bounds ${\bar{H}}_{\left(A_{p},A_{q}\right)}\left(D/A\right)$, $H_{\left(A_{p},A_{q}\right)}^{*}\left(D/A\right)$. An interesting question naturally emerges, i.e., can we necessarily determine the size relationship between ${\bar{H}}_{\left(A_{p},A_{q}\right)}\left(D/A\right)$ and $H_{\left(A_{p},A_{q}\right)}^{*}\left(D/A\right)$ to provide an exact bound? Unfortunately, the answer is negative, and the later example and experiment will reveal the size uncertainty. We simply provide a mechanism analysis. Let

$$P_{pq}=\frac{|A_{p}\cap D_{j}\left|+\right|A_{q}\cap D_{j}|}{|A_{p}\left|+\right|A_{q}|},$$

and its numerator/denominator be the corresponding sum of numerators/denominators of $P_{p}$ and $P_{q}$. According to [64], we can obtain

$$P_{pq}\in \left[min\left(P_{p},P_{q}\right),max\left(P_{p},P_{q}\right)\right]$$

but $P_{pq}$ produces an uncertainty location between $P_{p}$ and $P_{q}$. In view of the information function $f\left(P\right)=-P\log_{2}P$ and its maximum point $\left(\frac{1}{e},\frac{1}{e\ln2}\right)$ (Figure 2),

$$f\left(P_{p}\right)+f\left(P_{q}\right),f\left(P_{pq}\right)$$

never having the necessary size relationships, so

$${\bar{H}}_{\left(A_{p},A_{q}\right)}\left(D/A\right)=\sum_{j=1}^{m}\left(f\left(P_{p}\right)+f\left(P_{q}\right)\right),H_{\left(A_{p},A_{q}\right)}^{*}\left(D/A\right)=\sum_{j=1}^{m}f\left(P_{pq}\right)$$

also never have the necessary size relationships. In summary, ${\bar{H}}_{\left(A_{p},A_{q}\right)}\left(D/A\right)$ and $H_{\left(A_{p},A_{q}\right)}^{*}\left(D/A\right)$ adopt different views to become irrelevant and interactive, and they together restrict $H_{\left(A_{p},A_{q}\right)}\left(D/A\right)$. With the addition of lower bound of ${\underline{H}}_{\left(A_{p},A_{q}\right)}\left(D/A\right)$, there are in total three bounds to systematically emerge. Similar to $H_{\left(A_{p},A_{q}\right)}\left(D/A\right)$ and its distributional Table 2, they can also be arranged in a table with an n×n square symmetric matrix, i.e., Table 3, and thus Table 3 correspondingly restricts Table 2.

Finally, consider relevant granulation monotonicity/non-monotonicity. In fact, micro-bottom and its double-granule conditional-entropies focus on only two condition granules and thus never consider the condition granulation and further monotonicity/non-monotonicity. Moreover, U/IND(A)⪰U/IND(B) implies the granulation refining and granule decomposition from A to B; thus $A_{p},A_{q}\in U/IND\left(A\right)$ and $B_{p^{*}},B_{q^{*}}\in U/IND\left(B\right)$ exhibit complex correspondence and uncertainty change, so we cannot mine fine relationships between $H_{\left(A_{p},A_{q}\right)}\left(D/A\right)$ and $H_{\left(B_{p^{*}},B_{q^{*}}\right)}\left(D/B\right)$.

### 3.2. Double-Granule Conditional-Entropy at Meso-Middle

As analyzed above, double-granule conditional-entropies at micro-bottom never consider the condition granulation to lack robust functions of uncertainty descriptions. In terms of fixed decision granulation U/IND(D), $H_{\left(A_{p},A_{q}\right)}\left(D/A\right)$ at micro-bottom $\left(A_{p},A_{q}\right)$ involves only two condition granules $A_{p},A_{q}$ and their interactive uncertainty information. For the function promotion, the condition granulation U/IND(A) with systematic granules is worth introducing based on double-granule conditional-entropy $H_{\left(A_{p},A_{q}\right)}\left(D/A\right)$. Thus, we will gradually strengthen the knowledge granulation U/IND(A) to establish better double-granule conditional-entropies, by virtue of three-level granular structures (Table 1). This subsection discusses double-granule conditional-entropies at meso-middle

$$\left(A_{p},U/IND\left(A\right)=\left\{A_{1},\cdots ,A_{n}\right\}\right)\;\left(p\in \left\{1,\cdots ,n\right\}\right).$$

**Definition** **5.** *At meso-middle $\left(A_{p},U/IND\left(A\right)\right)$, the double-granule conditional-entropy is defined by*

(21) $$\begin{array}{cc} H_{\left(A_{p}\right)}\left(D/A\right) & =-\sum_{q=1}^{n}\left(\omega_{p}\sum_{j=1}^{m}P\left(D_{j}/A_{p}\right)\log_{2}P\left(D_{j}/A_{p}\right)\right)-\sum_{q=1}^{n}\left(\omega_{q}\sum_{j=1}^{m}P\left(D_{j}/A_{q}\right)\log_{2}P\left(D_{j}/A_{q}\right)\right)\\ & =-\sum_{q=1}^{n}\left(\frac{|A_{p}|}{|A_{p}\left|+\right|A_{q}|}\sum_{j=1}^{m}\frac{|A_{p}\cap D_{j}|}{|A_{p}|}\log_{2}\frac{|A_{p}\cap D_{j}|}{|A_{p}|}\right)-\sum_{q=1}^{n}\left(\frac{|A_{q}|}{|A_{p}\left|+\right|A_{q}|}\sum_{j=1}^{m}\frac{|A_{q}\cap D_{j}|}{|A_{q}|}\log_{2}\frac{|A_{q}\cap D_{j}|}{|A_{q}|}\right). \end{array}$$

**Corollary** **1.** *At meso-middle, the double-granule conditional-entropy has an analytic expression:*

(22) $$\begin{array}{c} H_{\left(A_{p}\right)}\left(D/A\right)=-\left(\sum_{q=1}^{n}\frac{|A_{p}|}{|A_{p}\left|+\right|A_{q}|}\right)\left(\sum_{j=1}^{m}P\left(D_{j}/A_{p}\right)\log_{2}P\left(D_{j}/A_{p}\right)\right)-\sum_{q=1}^{n}\left(\frac{|A_{q}|}{|A_{p}\left|+\right|A_{q}|}\sum_{j=1}^{m}P\left(D_{j}/A_{q}\right)\log_{2}P\left(D_{j}/A_{q}\right)\right). \end{array}$$

**Theorem** **4.** *Double-granule conditional-entropies have a hierarchical integration from micro-bottom to meso-middle, i.e.,*

(23) $$\begin{array}{c} H_{\left(A_{p}\right)}\left(D/A\right)=\sum_{q=1}^{n}H_{\left(A_{p},A_{q}\right)}\left(D/A\right)=H_{\left(A_{p},A_{1}\right)}\left(D/A\right)+\cdots +H_{\left(A_{p},A_{q}\right)}\left(D/A\right)+\cdots +H_{\left(A_{p},A_{n}\right)}\left(D/A\right). \end{array}$$

By Definition 5 (Corollary 1) and Theorem 4, meso-middle’s measure $H_{\left(A_{p}\right)}\left(D/A\right)$ (which can also be noted by $H_{\left(A_{p},U/IND\left(A\right)\right)}\left(D/A\right)$) hierarchically integrates double-granule conditional-entropies $H_{\left(A_{p},A_{q}\right)}\left(D/A\right)$ by condition-granular summation on q=1,⋯,n. Thus, $H_{\left(A_{p}\right)}\left(D/A\right)$ inherits the features of double-granule and conditional-entropy, it considers a granule $A_{p}$ and condition granulation U/IND(A) to be at meso-middle $\left(A_{p},U/IND\left(A\right)\right)$, so it is called the double-granule conditional-entropy at meso-middle. As a transition, $H_{\left(A_{p}\right)}\left(D/A\right)$ combines granule $A_{p}$ and partition U/IND(A) to describe decision classification U/IND(D) and its uncertainty, from the perspective of conditional information.

Similar to and based on previous discussions on $H_{\left(A_{p},A_{q}\right)}\left(D/A\right)$ (Section 3.1), we will provide corresponding properties of $H_{\left(A_{p}\right)}\left(D/A\right)$, including the number distribution, calculation algorithm, three bounds, and granulation monotonicity/non-monotonicity.

**Proposition** **3.** *At meso-middle, double-granule conditional-entropies offer n values, i.e.,*

$$H_{\left(A_{p}\right)}\left(D/A\right)\;\left(p=1,\cdots ,n\right).$$

In Proposition 3, double-granule conditional-entropies naturally exhibit number *n* to correspond to *n* meso-middles. The *n* values can be stored in an *n*-dimension vector to be related to the previous distributional Table 2. By enlarging Table 2, they are represented by the marginal vector of the bottom or right in Table 4, and they exactly correspond to the relevant row/column sum of micro-bottom’s information values. According to Equations (21) and (23), Algorithm 2 resorts to two “for” loops to effectively offer a double-granule conditional-entropy $H_{\left(A_{p}\right)}\left(D/A\right)$ for an arbitrary granule $A_{p}\;\in \;U/IND\left(A\right)$. In fact, the inner loop invokes Algorithm 1 to calculate an arbitrary double-granule conditional-entropy at micro-bottom, while the outer loop integrates *n* related bottomed measures to produce $H_{\left(A_{p}\right)}\left(D/A\right)$. Furthermore, we can achieve all *n* middle entropies values by adding a “for” loop regarding p=1,⋯,n.

**Algorithm 2:** Calculation of double-granule conditional-entropy at meso-middle**Input:** Decision table (U,C∪D), target subset A⊆C, and a special index p∈{1,⋯,n}; **Output:** Double-granule conditional-entropy $H_{\left(A_{p}\right)}\left(D/A\right)$ at meso-middle $\left(A_{p},U/IND\left(A\right)\right)$.
  1:Compute U/IND(A) to obtain all condition classes $A_{i}$ (i=1,⋯,n) and a fixed granule $A_{p}\in U/IND\left(A\right)$.  2:Compute U/IND(D) to obtain all decision classes $D_{j}$ (j=1,⋯,m).  3:Let $H_{\left(A_{p}\right)}\left(D/A\right)=0$.  4:**for**q∈{1,..,n}**do**  5:  Compute $\omega_{p},\omega_{q}$.  6:  Let $H_{p}=0$, $H_{q}=0$.  7:  **for** j∈{1,..,m} **do**  8:   $H_{p}\leftarrow H_{p}-P\left(D_{j}/A_{p}\right)\log_{2}P\left(D_{j}/A_{p}\right)$,   $H_{q}\leftarrow H_{q}-P\left(D_{j}/A_{q}\right)\log_{2}P\left(D_{j}/A_{q}\right)$.  9:  **end for**10:  Obtain $H_{\left(A_{p},A_{q}\right)}\left(D/A\right)=\omega_{p}H_{p}+\omega_{q}H_{q}$.11:  $H_{\left(A_{p}\right)}\left(D/A\right)\leftarrow H_{\left(A_{p}\right)}\left(D/A\right)+H_{\left(A_{p},A_{q}\right)}\left(D/A\right)$.12:**end for**13:**return**$H_{\left(A_{p}\right)}\left(D/A\right)$.

**Theorem** **5.** *At meso-middle, the double-granule conditional-entropy has a lower bound and two upper bounds. Concretely,*

(24) $$\begin{array}{c} \begin{array}{cc} H_{\left(A_{p}\right)}\left(D/A\right) & \in [{\underline{H}}_{\left(A_{p}\right)}\left(D/A\right),{\bar{H}}_{\left(A_{p}\right)}\left(D/A\right)],\\ H_{\left(A_{p}\right)}\left(D/A\right) & \leq H_{\left(A_{p}\right)}^{*}\left(D/A\right), \end{array} \end{array}$$

*where*

(25) $$\begin{array}{cc} {\underline{H}}_{\left(A_{p}\right)}\left(D/A\right) & =\sum_{q=1}^{n}{\underline{H}}_{\left(A_{p},A_{q}\right)}\left(D/A\right)=-\frac{n|A_{p}|}{2|U|}\sum_{j=1}^{m}P\left(D_{j}/A_{p}\right)\log_{2}P\left(D_{j}/A_{p}\right)-\sum_{q=1}^{n}\left(\frac{|A_{q}|}{2|U|}\sum_{j=1}^{m}P\left(D_{j}/A_{q}\right)\log_{2}P\left(D_{j}/A_{q}\right)\right),\\ {\bar{H}}_{\left(A_{p}\right)}\left(D/A\right) & =\sum_{q=1}^{n}{\bar{H}}_{\left(A_{p},A_{q}\right)}\left(D/A\right)=-n\sum_{j=1}^{m}P\left(D_{j}/A_{p}\right)\log_{2}P\left(D_{j}/A_{p}\right)-\sum_{q=1}^{n}\sum_{j=1}^{m}P\left(D_{j}/A_{q}\right)\log_{2}P\left(D_{j}/A_{q}\right),\\ H_{\left(A_{p}\right)}^{*}\left(D/A\right) & =\sum_{q=1}^{n}H_{\left(A_{p},A_{q}\right)}^{*}\left(D/A\right)=-\sum_{q=1}^{n}\sum_{j=1}^{m}\frac{|A_{p}\cap D_{j}\left|+\right|A_{q}\cap D_{j}|}{|A_{p}\left|+\right|A_{q}|}\log_{2}\frac{|A_{p}\cap D_{j}\left|+\right|A_{q}\cap D_{j}|}{|A_{p}\left|+\right|A_{q}|}. \end{array}$$

Theorem 5 naturally comes from Theorems 2–4. The three bounds in Equation (25) hierarchically integrate previous three bounds at micro-bottom (Equations (11) and (17)) to correspondingly restrict $H_{\left(A_{p}\right)}\left(D/A\right)$. They can be supplemented into distributional Table 4, and following Table 5 provides the relevant part.

At meso-middle, $H_{\left(A_{p}\right)}\left(D/A\right)$ introduces the condition granulation U/IND(A), but it still needs condition granule $A_{p}$. Thus, we cannot make a positive assertion regarding granulation monotonicity/non-monotonicity. In fact, U/IND(A)⪰U/IND(B) also implies chaos between $H_{\left(A_{p}\right)}\left(D/A\right)$ and $H_{\left(B_{p^{*}}\right)}\left(D/B\right)$.

### 3.3. Double-Granule Conditional-Entropy at Macro-Top

As analyzed above, double-granule conditional-entropies at meso-middle consider the condition granulation, but in an insufficient way, and $H_{\left(A_{p}\right)}\left(D/A\right)$ also depends on a single condition granule $A_{p}$. For the thorough granulation and robust description, systematic measures $H_{\left(A_{p}\right)}\left(D/A\right)$ (p=1,⋯,n) can be further integrated to generate double-granule conditional-entropies at macro-top. Based on the previous thought and result in Section 3.1 and Section 3.2, this subsection further discusses double-granule conditional-entropies at macro-top

$$(U/IND\left(A\right)=\left\{A_{p}:p=1,\cdots ,n\right\},U/IND\left(A\right)=\left\{A_{q}:q=1,\cdots ,n\right\}),$$

which is given in Table 1. We will directly provide the relevant integration definition, number distribution, calculation algorithm, three bounds, and we finally uncover an important conclusion of granulation non-monotonicity.

**Definition** **6.** *At macro-top (U/IND(A),U/IND(A)), the double-granule conditional-entropy is defined by*

(26) $$\begin{array}{cc} H(D/A) & =-\sum_{p=1}^{n}\sum_{q=1}^{n}\left(\omega_{p}\sum_{j=1}^{m}P\left(D_{j}/A_{p}\right)\log_{2}P\left(D_{j}/A_{p}\right)\right)-\sum_{p=1}^{n}\sum_{q=1}^{n}\left(\omega_{q}\sum_{j=1}^{m}P\left(D_{j}/A_{q}\right)\log_{2}P\left(D_{j}/A_{q}\right)\right)\\ & =-\sum_{p=1}^{n}\sum_{q=1}^{n}\left(\frac{|A_{p}|}{|A_{p}\left|+\right|A_{q}|}\sum_{j=1}^{m}\frac{|A_{p}\cap D_{j}|}{|A_{p}|}\log_{2}\frac{|A_{p}\cap D_{j}|}{|A_{p}|}\right)-\sum_{p=1}^{n}\sum_{q=1}^{n}\left(\frac{|A_{q}|}{|A_{p}\left|+\right|A_{q}|}\sum_{j=1}^{m}\frac{|A_{q}\cap D_{j}|}{|A_{q}|}\log_{2}\frac{|A_{q}\cap D_{j}|}{|A_{q}|}\right). \end{array}$$

**Corollary** **2.** *At macro-top, the double-granule conditional-entropy has an analytic expression:*

(27) $$\begin{array}{c} H\left(D/A\right)=-\sum_{p=1}^{n}\left(\sum_{q=1}^{n}\frac{|A_{p}|}{|A_{p}\left|+\right|A_{q}|}\right)\left(\sum_{j=1}^{m}P\left(D_{j}/A_{p}\right)\log_{2}P\left(D_{j}/A_{p}\right)\right)-\sum_{p=1}^{n}\sum_{q=1}^{n}\left(\frac{|A_{q}|}{|A_{p}\left|+\right|A_{q}|}\sum_{j=1}^{m}P\left(D_{j}/A_{q}\right)\log_{2}P\left(D_{j}/A_{q}\right)\right). \end{array}$$

**Theorem** **6.** *Double-granule conditional-entropies have a hierarchical integration from micro-bottom and meso-middle to macro-top, i.e.,*

(28) $$\begin{array}{c} \begin{array}{cc} H(D/A) & =\sum_{p=1}^{n}H_{\left(A_{p}\right)}\left(D/A\right)=H_{\left(A_{1}\right)}\left(D/A\right)+\cdots +H_{\left(A_{n}\right)}\left(D/A\right)\\ & =\sum_{p=1}^{n}\sum_{q=1}^{n}H_{\left(A_{p},A_{q}\right)}\left(D/A\right)=H_{\left(A_{1},A_{1}\right)}\left(D/A\right)+\cdots +H_{\left(A_{n},A_{n}\right)}\left(D/A\right). \end{array} \end{array}$$

By Definition 6 (Corollary 2) and Theorem 6, macro-top’s measure H(D/A) hierarchically integrates meso-middle’s entropies $H_{\left(A_{p}\right)}\left(D/A\right)$ by a single summation on p=1,⋯,n, and thus it further hierarchically integrates micro-bottom’s entropies $H_{\left(A_{p},A_{q}\right)}\left(D/A\right)$ by double summations on p,q=1,⋯,n. As a result, H(D/A) inherits the features of double-granule and conditional-entropy. It considers only conditional granulation U/IND(A) to be at macro-top (U/IND(A),U/IND(A)), so it is called the double-granule conditional-entropy at macro-top. As an ultimate measure, H(D/A) completely utilizes the U/IND(A) granulation information to effectively describe decision classification U/IND(D) and its uncertainty, thus holding robust measurement functions for knowledge granulation. Moreover, H(D/A) can be noted by $H_{\left(U/IND(A),U/IND(A)\right)}\left(D/A\right)$).

**Proposition** **4.** *At macro-top, the double-granule conditional-entropy offers only one value, i.e.,*

H(D/A) at macro-top (U/IND(A),U/IND(A)).

In Proposition 4, the double-granule conditional-entropy naturally exhibits number 1 to correspond to the sole macro-top. In fact, the first top entropy comes from the fusion of either *n* middle entropies or n×n bottom entropies; thus, three-level entropies accord with three-level granular structures (Table 1) from the quantitative and structural perspective, and they embody the hierarchical integration. In particular, the sole conditional-entropy H(D/A) is put into the lower-right corner of Table 4, thus corresponding to the summations of central n×n micro values and marginal *n* meso values. According to Equations (26) and (28), Algorithm 3 resorts to three “for” loops to effectively offer the double-granule conditional-entropy H(D/A). The two inner loops invoke Algorithm 2 to calculate an arbitrary double-granule conditional-entropy at meso-middle (where the central loop invokes Algorithm 1 to construct micro-bottom’s entropies), while the outer loop integrates *n* related meso-middle’s information values to produce H(D/A). In other words, Algorithms 1–3 exhibit a kind of hierarchical evolution based on circulation development, and thus they constitute a novel kind of three-level algorithms.

**Algorithm 3:** Calculation of double-granule conditional-entropy at macro-top**Input:** Decision table (U,C∪D), target subset A⊆C; **Output:** Double-granule conditional-entropy H(D/A) at Macro-Top (U/IND(A),U/IND(A)).
  1:Compute U/IND(A) to obtain all condition classes $A_{i}$ (i=1,⋯,n).  2:Compute U/IND(D) to obtain all decision classes $D_{j}$ (j=1,⋯,m).  3:Let H(D/A)=0.  4:**for**p∈{1,..,n}**do**  5:  Let $H_{\left(A_{p}\right)}\left(D/A\right)=0$.  6:  **for** q∈{1,..,n} **do**  7:   Compute $\omega_{p},\omega_{q}$.  8:   Let $H_{p}=0$, $H_{q}=0$.  9:   **for** j∈{1,..,m} **do**10:    $H_{p}\leftarrow H_{p}-P\left(D_{j}/A_{p}\right)\log_{2}P\left(D_{j}/A_{p}\right)$,    $H_{q}\leftarrow H_{q}-P\left(D_{j}/A_{q}\right)\log_{2}P\left(D_{j}/A_{q}\right)$.11:   **end for**12:   Obtain $H_{\left(A_{p},A_{q}\right)}\left(D/A\right)=\omega_{p}H_{p}+\omega_{q}H_{q}$.13:   $H_{\left(A_{p}\right)}\left(D/A\right)\leftarrow H_{\left(A_{p}\right)}\left(D/A\right)+H_{\left(A_{p},A_{q}\right)}\left(D/A\right)$.14:  **end for**15:  $H\left(D/A\right)\leftarrow H\left(D/A\right)+H_{\left(A_{p}\right)}\left(D/A\right)$.16:**end for**17:**return**H(D/A).

**Theorem** **7.** *At macro-top, the double-granule conditional-entropy has a lower bound and two upper bounds. Concretely,*

(29) $$\begin{array}{c} \begin{array}{cc} H(D/A) & \in [\underline{H}\left(D/A\right),\bar{H}\left(D/A\right)],\\ H(D/A) & \leq H^{*}\left(D/A\right), \end{array} \end{array}$$

*where*

(30) $$\begin{array}{cc} \underline{H}\left(D/A\right) & =\sum_{p=1}^{n}{\underline{H}}_{\left(A_{p}\right)}\left(D/A\right)=\sum_{p=1}^{n}\sum_{q=1}^{n}{\underline{H}}_{\left(A_{p},A_{q}\right)}\left(D/A\right)\\ & =-n\sum_{p=1}^{n}\left(\frac{|A_{p}|}{2|U|}\sum_{j=1}^{m}P\left(D_{j}/A_{p}\right)\log_{2}P\left(D_{j}/A_{p}\right)\right)-n\sum_{q=1}^{n}\left(\frac{|A_{q}|}{2|U|}\sum_{j=1}^{m}P\left(D_{j}/A_{q}\right)\log_{2}P\left(D_{j}/A_{q}\right)\right)\\ & =-n\sum_{p=1}^{n}\left(\frac{|A_{p}|}{\left|U\right|}\sum_{j=1}^{m}P\left(D_{j}/A_{p}\right)\log_{2}P\left(D_{j}/A_{p}\right)\right), \end{array}$$

(31) $$\begin{array}{c} \begin{array}{cc} \bar{H}\left(D/A\right) & =\sum_{p=1}^{n}{\bar{H}}_{\left(A_{p}\right)}\left(D/A\right)=\sum_{p=1}^{n}\sum_{q=1}^{n}{\bar{H}}_{\left(A_{p},A_{q}\right)}\left(D/A\right)\\ & =-n\sum_{p=1}^{n}\sum_{j=1}^{m}P\left(D_{j}/A_{p}\right)\log_{2}P\left(D_{j}/A_{p}\right)-n\sum_{q=1}^{n}\sum_{j=1}^{m}P\left(D_{j}/A_{q}\right)\log_{2}P\left(D_{j}/A_{q}\right)\\ & =-2n\sum_{p=1}^{n}\sum_{j=1}^{m}P\left(D_{j}/A_{p}\right)\log_{2}P\left(D_{j}/A_{p}\right), \end{array} \end{array}$$

(32) $$\begin{array}{c} \begin{array}{cc} H^{*}\left(D/A\right) & =\sum_{p=1}^{n}H_{\left(A_{p}\right)}^{*}\left(D/A\right)=\sum_{p=1}^{n}\sum_{q=1}^{n}H_{\left(A_{p},A_{q}\right)}^{*}\left(D/A\right)\\ & =-\sum_{p=1}^{n}\sum_{q=1}^{n}\sum_{j=1}^{m}\frac{|A_{p}\cap D_{j}\left|+\right|A_{q}\cap D_{j}|}{|A_{p}\left|+\right|A_{q}|}\log_{2}\frac{|A_{p}\cap D_{j}\left|+\right|A_{q}\cap D_{j}|}{|A_{p}\left|+\right|A_{q}|}. \end{array} \end{array}$$

Theorem 7 naturally comes from Theorems 2–6. The three bounds in Equations (30)–(32) hierarchically integrate previous three bounds at meso-middle and micro-bottom, and thus they become three new uncertainty measures at macro-top (U/IND(A),U/IND(A)) to correspondingly restrict H(D/A). They are supplemented into the bottom in the previous bound table: Table 5.

**Theorem** **8.** *At macro-top, the double-granule conditional-entropy has granulation non-monotonicity. That is, U/IND(A)⪰U/IND(B) cannot necessarily achieve*

either H(D/A)≥H(D/B) or H(D/A)≤H(D/B),

*and both cases can practically exist. In addition, the matched double bounds $\underline{H}\left(D/A\right)$ and $\bar{H}\left(D/A\right)$ (Equations (30) and (31)) also have the granulation non-monotonicity, and they cannot theoretically acquire*

$$\mathrm{either}\;\underline{H}\left(D/A\right)\geq \underline{H}\left(D/B\right)\;\mathrm{or}\;\underline{H}\left(D/A\right)\leq \underline{H}\left(D/B\right),$$

$$\mathrm{either}\;\bar{H}\left(D/A\right)\geq \bar{H}\left(D/B\right)\;\mathrm{or}\;\bar{H}\left(D/A\right)\leq \bar{H}\left(D/B\right).$$

At macro-top, the double-granule conditional-entropy completely breaks away from the condition granule dependence to establish the condition granulation description, so it becomes a powerful type of information measure for knowledge-based uncertainty representation. In terms of condition granulation, its non-monotonicity is finally revealed in Theorem 8, and the relevant evidence will be provided in the later example and experiment. Moreover, this fundamental non-monotonicity conclusion embodies information uncertainty, and it can be induced or explained by the previous complexity mechanism at micro-bottom and meso-middle. Based on macro-top and its granulation mechanism, the related three bounds (Equations (30)–(32)) and their monotonicity/non-monotonicity can be practically observed, and thus we also obtain the granulation non-monotonicity for $\underline{H}\left(D/A\right)$ and $\bar{H}\left(D/A\right)$; however, the case of upper bound $H^{*}\left(D/A\right)$ becomes a remaining problem.

## 4. Decision Table Example

In this section, the above theoretical constructions and properties are illustrated by a decision table example. By extracting a part of VOTING data set (which comes from UCI database [65]), we provide a practical decision table (U,C∪D) in Table 6 with

|U|=8,|C|=11,|D|=1.

According to this decision table,

$$U/IND\left(D\right)=\{D_{1}=\left\{x_{1},x_{2},x_{8}\right\},D_{2}=\left\{x_{3},x_{4},x_{5},x_{6},x_{7}\right\}\}$$

provides m=|U/IND(D)|=2. As an example, $A=\{c_{1},c_{2},c_{3},c_{4},c_{5}\}$ is chosen to generate condition granulation

$$U/IND\left(A\right)=\{A_{1}=\left\{x_{1}\right\},A_{2}=\left\{x_{2},x_{7},x_{8}\right\},A_{3}=\left\{x_{3}\right\},A_{4}=\left\{x_{4}\right\},A_{5}=\left\{x_{5}\right\},A_{6}=\left\{x_{6}\right\}\},$$

where n=|U/IND(A)|=6. By virtue of three-level granular structures (Table 1), double-granule conditional-entropies and their three bounds are calculated by relevant algorithms and definitions, and they are compactly listed in Table 7 and Table 8, respectively. The measures at micro-bottom, meso-middle, macro-top have numbers 36, 6, 1, respectively, and they correspond to the central 6×6 matrix, marginal 6-dimensional vector, lower-right-corner 1 digit, respectively. In part, we provide some processes of entropy calculation as follows.

(33) $$\begin{array}{c} \begin{array}{cc} -P\left(D_{1}/A_{1}\right)\log_{2}P\left(D_{1}/A_{1}\right) & =0,-P\left(D_{2}/A_{1}\right)\log_{2}P\left(D_{2}/A_{1}\right)=0,\\ -P\left(D_{1}/A_{2}\right)\log_{2}P\left(D_{1}/A_{2}\right) & =0.3900,-P\left(D_{2}/A_{2}\right)\log_{2}P\left(D_{2}/A_{2}\right)=0.5283,\\ H_{\left(A_{1},A_{1}\right)}\left(D/A\right) & =\frac{1}{1+1}\left(0+0\right)+\frac{1}{1+1}\left(0+0\right)=0,\\ H_{\left(A_{1},A_{2}\right)}\left(D/A\right) & =\frac{1}{1+3}\left(0+0\right)+\frac{3}{1+3}\left(0.3900+0.5283\right)=0.6887;\\ H_{\left(A_{1}\right)}\left(D/A\right) & =H_{\left(A_{1},A_{1}\right)}\left(D/A\right)+H_{\left(A_{1},A_{2}\right)}\left(D/A\right)+\cdots +H_{\left(A_{1},A_{6}\right)}\left(D/A\right)\\ & =0+0.6887+0+0+0+0=0.6887;\\ H(D/A) & =H_{\left(A_{1}\right)}\left(D/A\right)+H_{\left(A_{2}\right)}\left(D/A\right)+\cdots +H_{\left(A_{6}\right)}\left(D/A\right)\\ & =0.6887+4.3619+0.6887+0.6887+0.6887+0.6887=7.8055. \end{array} \end{array}$$

By Table 7 and Table 8, we can make relevant verification analyses. First, entropies and bounds naturally present hierarchical integration relationships from micro-bottom to meso-middle to macro-top. Indeed, conditional-entropies are correspondingly restricted by three bounds. Moreover, the two types of upper bounds exactly have no necessary size relationships, and a part but powerful proof is provided as follows:

$$\begin{array}{c} \left\{\begin{array}{c} \;{\bar{H}}_{\left(A_{1},A_{2}\right)}\left(D/A\right)=0.9183>0.8113=H_{\left(A_{1},A_{2}\right)}^{*}\left(D/A\right),\\ \;{\bar{H}}_{\left(A_{1},A_{3}\right)}\left(D/A\right)=0<1=H_{\left(A_{1},A_{3}\right)}^{*}\left(D/A\right); \end{array}\right. \end{array}$$

$$\begin{array}{c} \left\{\begin{array}{c} \;{\bar{H}}_{\left(A_{1}\right)}\left(D/A\right)=0.9183<4.8113=H_{\left(A_{1}\right)}^{*}\left(D/A\right),\\ \;{\bar{H}}_{\left(A_{2}\right)}\left(D/A\right)=6.4281>5.7296=H_{\left(A_{2}\right)}^{*}\left(D/A\right). \end{array}\right. \end{array}$$

Finally, the granulation non-monotonicity at macro-top (Theorem 8) is verified. For this, we chose a natural attribute-addition chain:

$$\left\{c_{1}\right\}\subset \left\{c_{1},c_{2}\right\}\subset \cdots \subset \left\{c_{1},c_{2},\cdots ,c_{11}\right\}.$$

$CA_{k}$ (k∈{1,2,⋯,11}) denotes the attribute subset in the chain, and its granulation is represented by

$$U/IND\left(CA_{k}\right)=\left\{CA_{k,1},\cdots ,CA_{k,p},\cdots ,CA_{k,|U/IND(CA_{k})|}\right\}.$$

In other words, $CA_{k,p}$ corresponds to the kth chain element $CA_{k}$ to represent the pth condition granule in partition $U/IND(CA_{k})$. According to the subset chain, Table 9 provides double-granule conditional-entropies, including both part values at micro-bottom $\left(CA_{k,p},CA_{k,q}\right)$, meso-middle $\left(CA_{k,p},U/IND\left(CA_{k}\right)\right)$ and all values (as well as the three bounds) at macro-top $\left(U/IND\left(CA_{k}\right),U/IND\left(CA_{k}\right)\right)$. As a supporting detail, previous Table 7 and Table 8 actually embrace the chain element $CA_{5}$ and its partition $U/IND\left(CA_{5}\right)=\left\{\left\{x_{1}\right\},\left\{x_{2},x_{7},x_{8}\right\},\left\{x_{3}\right\},\left\{x_{4}\right\},\left\{x_{5}\right\},\left\{x_{6}\right\}\right\}$, while double-granule conditional-entropies regarding attribute subset $CA_{2}=\left\{c_{1},c_{2}\right\}$ and corresponding condition granulation

$$U/IND\left(CA_{2}\right)=\left\{CA_{2,1}=\left\{x_{1},x_{2},x_{7},x_{8}\right\},CA_{2,2}=\left\{x_{3}\right\},CA_{2,3}=\left\{x_{4},x_{6}\right\},CA_{2,4}=\left\{x_{5}\right\}\right\}$$

are supplemented in Table 10 for better observation and illustration.

**Figure 3:**
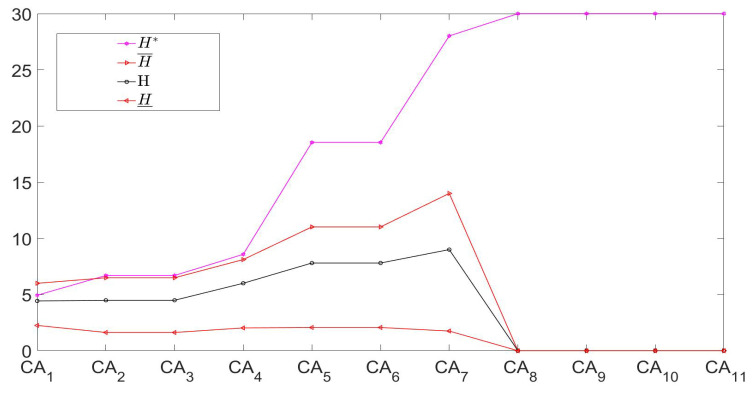
Macro-top’s double-granule conditional-entropies and their three bounds based on an attribute-enlargement chain in the example.

(1) Since different chain subsets may have different equivalence partitions and granule numbers, the measures at micro-bottom and meso-middle consider condition granules to have a distinctive number and difficult correspondence. Table 9 focuses on the small and the same granule number, but relevant granules have different connotations. For example, the granules of the first one —$CA_{k,1}$ (k=1,2,⋯,11)—may be different. Thus, we cannot acquire the so-called granulation non-monotonicity assertion because of granulation incompletion, although the values at micro-bottom and meso-middle actually exhibit a kind of non-monotonic change in Table 9.
(2) In contrast, macro-top offers the complete condition granulation, so we can effectively focus on value monotonicity/non-monotonicity for both double-granule conditional-entropies and their three bounds. Observing the bottom part of Table 9 in the enlargement chain direction, we can discover that the three types of information measures are all non-monotonic, i.e.,
  $$H(D/CA_{k}),\underline{H}\left(D/CA_{k}\right),\bar{H}\left(D/CA_{k}\right)(\mathrm{except}\;H^{*}\left(D/CA_{k}\right)).$$
  More vividly, the entropy and its three bounds regarding the chain are depicted in Figure 3, so the related granulation non-monotonicity becomes clearer. For example, the macro entropy value $H(D/CA_{k})$ first increases and then decreases in the addition chain direction. Moreover, Table 9 and Figure 3 reflect the restriction properties of three bounds.

## 5. Data Experiments

In this section, the above theoretical results and their effectiveness are verified by data experiments. The new measures are mainly suitable for categorical (or nominal) data, which are usually used in the traditional rough set theory, and thus we adopt three relevant data sets from the UCI Machine Learning Repository [65], whose concrete descriptions on decision table (U,C∪D) are given in Table 11.

Similar to the above example, we also adopt the attribute-addition chain

(34) $$\begin{array}{c} \begin{array}{c} CA_{1}=\left\{c_{1}\right\}\subset \cdots \subset CA_{\left|C\right|}=\left\{c_{1},c_{2},\cdots \;c_{\left|C\right|}\right\} \end{array} \end{array}$$

and its relevant symbol such as

$$U/IND\left(CA_{k}\right)=\left\{CA_{k,1},\cdots ,CA_{k,p},\cdots ,CA_{k,|U/IND(CA_{k})|}\right\}.$$

Note that this attribute-subset sequence (Equation (34)) can deeply and typically probe the hierarchical knowledge-granulation within a framework of the complete lattice $\left(2^{C},\subseteq \right)$. As a representative manifestation, we provide two typical results regarding the first chain element $CA_{1}=\left\{c_{1}\right\}$ and the last one $CA_{\left|C\right|}=C$.

(1) Regarding VOTING, $\left\{c_{1}\right\}$ and C induce three and 342 granules, respectively, and relevant double-granule conditional-entropies and three bounds are provided in Table 12 and Table 13, respectively.
(2) Regarding SPECT, $\left\{c_{1}\right\}$ and C produce two and 169 granules, respectively, and relevant three-level measures and three bounds are provided in Table 14 and Table 15, respectively.
(3) Regarding Tic-Tac-Toe, $\left\{c_{1}\right\}$ and C determine three and 958 granules, respectively, and relevant entropies and bounds are provided in Table 16 and Table 17, respectively.

**Figure 4:**
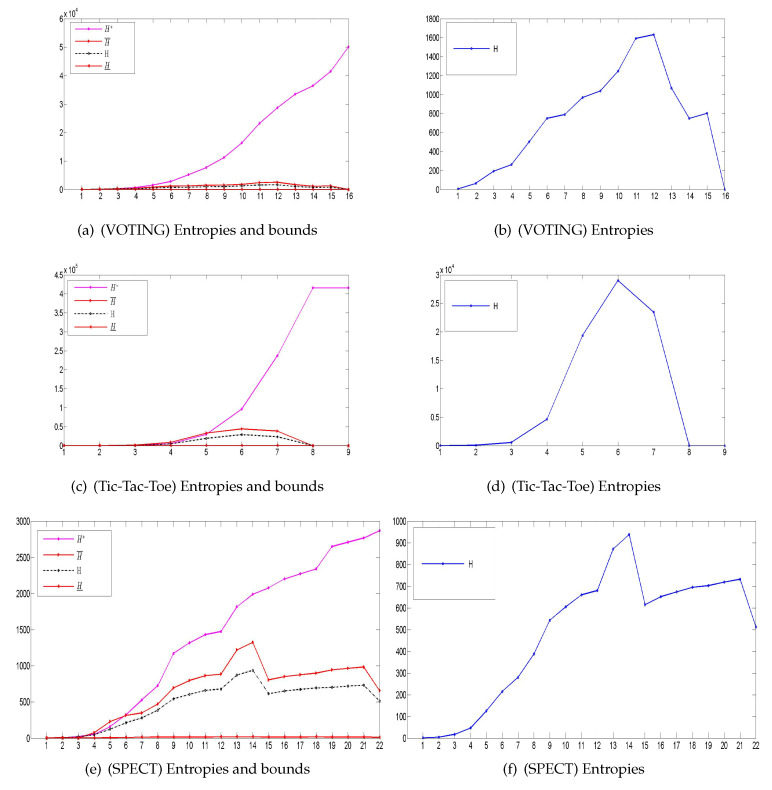
Macro-top’s double-granule conditional-entropies and their three information bounds based on an attribute-enlargement chain in data experiments.

From the perspective of macro-top, double-granule conditional-entropies and their three information bounds based on the attribute-enlargement chain are finally summarized in Figure 4. These tables and figures can be utilized to effectively verify all previous conclusions, including the hierarchy, algorithm, restriction, and non-monotonicity. In particular, double-granule conditional-entropies are confined by three bounds, thus supporting the boundedness (Theorems 2, 3, 5 and 7); moreover, the entropies and their matched double-bounds fluctuate up and down, thus proving relevant granulation non-monotonicity (Theorem 8).

## 6. Conclusions

The information measures implement fundamental uncertainty measurement in rough set theory and granular computing. The local conditional-entropies have the second-order feature, but they are limited to micro-bottom for describing discernibility matrix and reduction core [18]. In this paper, double-granule conditional-entropies achieve corresponding improvements of hierarchical/conditional granulation, and thus they become broader measures with uncertainty representation and information processing. They focus more on the double-granule interaction rather than granule-union locality, which is used in local conditional-entropies [18]. This strategy directly utilizes the second-order mechanism to implement more systematic and robust uncertainty measurements, especially when compared to the current mainstream of first-order information measures. In our studies, double-granule conditional-entropies and their hierarchies, granulation, algorithms, bounds, and non-monotonicity are acquired and verified at three-level granular structures (i.e., micro-bottom, meso-middle, macro-top), and these results underlie both the efficiency in information processing and effectiveness in knowledge-based data analyses. Furthermore, their future developments and in-depth applications can be explored as follows.

(1) In contrast to the relevant technology in [56], the hierarchical granulation of three-level granular structures focuses on the conditional granulation and relevant number, and it can be generalized for granular computing.
(2) The double-granule conditional-entropies and their three bounds become new types of information measures with the second-order feature. In contrast to the traditional first-order entropy system, their description power and application advantage need further practical verification.
(3) The double-granule conditional-entropies have three-restrictive bounds and granulation non-monotonicity, which have been experimentally verified by a granulation-hierarchical sequence (i.e., Equation (34)). These results are worth deeply utilizing in uncertainty measurement and data mining.
(4) The double-granule conditional-entropies originate from the local conditional-entropies to carry a potential and distinctive advantage of discernibility matrix representation, and they also have the complete conditional granulation to have application prospects in knowledge reasoning or acquisition. Both their relationships with the discernibility matrix and their functions on attribute reduction need be deeply researched by promoting the previous studies in [18].

## Figures and Tables

**Table 1:** Three-level granular structures based on condition granulation of the decision table.

Structure Naming	Composition System	Granular Scale	Granular Level	Number of Parallel Patterns
Micro-Bottom	$\left(A_{p},A_{q}\right)$	Micro	Bottom	n×n
Meso-Middle	$\left(A_{p},U/IND\left(A\right)\right)$ $=(A_{p},\left\{A_{q}:q=1,\cdots ,n\right\})$	Meso	Middle	*n*
Macro-Top	(U/IND(A),U/IND(A)) $=(\left\{A_{p}:p=1,\cdots ,n\right\},\left\{A_{q}:q=1,\cdots ,n\right\})$	Macro	Top	1

**Table 2:** Matrix distribution of double-granule conditional-entropies at micro-bottom.

U/IND(A)	$A_{1}$	⋯	$A_{q}$	⋯	$A_{n}$
$A_{1}$	$H_{\left(A_{1},A_{1}\right)}\left(D/A\right)$	⋯	$H_{\left(A_{1},A_{q}\right)}\left(D/A\right)$	⋯	$H_{\left(A_{1},A_{n}\right)}\left(D/A\right)$
⋮	⋮	⋱	⋮	⋱	⋮
$A_{p}$	$H_{\left(A_{p},A_{1}\right)}\left(D/A\right)$	⋯	$H_{\left(A_{p},A_{q}\right)}\left(D/A\right)$	⋯	$H_{\left(A_{p},A_{n}\right)}\left(D/A\right)$
⋮	⋮	⋱	⋮	⋱	⋮
$A_{n}$	$H_{\left(A_{n},A_{1}\right)}\left(D/A\right)$	⋯	$H_{\left(A_{n},A_{q}\right)}\left(D/A\right)$	⋯	$H_{\left(A_{n},A_{n}\right)}\left(D/A\right)$

**Table 3:** Three bounds of double-granule conditional-entropies at micro-bottom.

U/IND(A)	$A_{1}$	⋯	$A_{q}$	⋯	$A_{n}$
$A_{1}$	$\left[{\underline{H}}_{\left(A_{1},A_{1}\right)\left(D/A\right)}\left(D/A\right),{\bar{H}}_{\left(A_{1},A_{1}\right)}\left(D/A\right)\right]$ $H_{\left(A_{1},A_{1}\right)}^{*}\left(D/A\right)$	⋯	$\left[{\underline{H}}_{\left(A_{1},A_{q}\right)}\left(D/A\right),{\bar{H}}_{\left(A_{1},A_{q}\right)}\left(D/A\right)\right]$ $H_{\left(A_{1},A_{q}\right)}^{*}\left(D/A\right)$	⋯	$\left[{\underline{H}}_{\left(A_{1},A_{n}\right)}\left(D/A\right),{\bar{H}}_{\left(A_{1},A_{n}\right)}\left(D/A\right)\right]$ $H_{\left(A_{1},A_{n}\right)}^{*}\left(D/A\right)$
⋮	⋮	⋱	⋮	⋱	⋮
$A_{p}$	$\left[{\underline{H}}_{\left(A_{p},A_{1}\right)}\left(D/A\right),{\bar{H}}_{\left(A_{p},A_{1}\right)}\left(D/A\right)\right]$ $H_{\left(A_{p},A_{1}\right)}^{*}\left(D/A\right)$	⋯	$\left[{\underline{H}}_{\left(A_{p},A_{q}\right)}\left(D/A\right),{\bar{H}}_{\left(A_{p},A_{q}\right)}\left(D/A\right)\right]$ $H_{\left(A_{p},A_{q}\right)}^{*}\left(D/A\right)$	⋯	$\left[{\underline{H}}_{\left(A_{p},A_{n}\right)}\left(D/A\right),{\bar{H}}_{\left(A_{p},A_{n}\right)}\left(D/A\right)\right]$ $H_{\left(A_{p},A_{n}\right)}^{*}\left(D/A\right)$
⋮	⋮	⋱	⋮	⋱	⋮
$A_{n}$	$\left[{\underline{H}}_{\left(A_{n},A_{1}\right)}\left(D/A\right),{\bar{H}}_{\left(A_{n},A_{1}\right)}\left(D/A\right)\right]$ $H_{\left(A_{n},A_{1}\right)}^{*}\left(D/A\right)$	⋯	$\left[{\underline{H}}_{\left(A_{n},A_{q}\right)}\left(D/A\right),{\bar{H}}_{\left(A_{n},A_{q}\right)}\left(D/A\right)\right]$ $H_{\left(A_{n},A_{q}\right)}^{*}\left(D/A\right)$	⋯	$\left[{\underline{H}}_{\left(A_{n},A_{n}\right)}\left(D/A\right),{\bar{H}}_{\left(A_{n},A_{n}\right)}\left(D/A\right)\right]$ $H_{\left(A_{n},A_{n}\right)}^{*}\left(D/A\right)$

**Table 4:** Marginal distribution of double-granule conditional-entropies at meso-middle and macro-top.

U/IND(A)	$A_{1}$	⋯	$A_{q}$	⋯	$A_{n}$	Meso-Middle
$A_{1}$	$H_{\left(A_{1},A_{1}\right)}\left(D/A\right)$	⋯	$H_{\left(A_{1},A_{q}\right)}\left(D/A\right)$	⋯	$H_{\left(A_{1},A_{n}\right)}\left(D/A\right)$	$H_{\left(A_{1}\right)}\left(D/A\right)$
⋮	⋮	⋱	⋮	⋱	⋮	⋮
$A_{p}$	$H_{\left(A_{p},A_{1}\right)}\left(D/A\right)$	⋯	$H_{\left(A_{p},A_{q}\right)}\left(D/A\right)$	⋯	$H_{\left(A_{p},A_{n}\right)}\left(D/A\right)$	$H_{\left(A_{p}\right)}\left(D/A\right)$
⋮	⋮	⋱	⋮	⋱	⋮	⋮
$A_{n}$	$H_{\left(A_{n},A_{1}\right)}\left(D/A\right)$	⋯	$H_{\left(A_{n},A_{q}\right)}\left(D/A\right)$	⋯	$H_{\left(A_{n},A_{n}\right)}\left(D/A\right)$	$H_{\left(A_{n}\right)}\left(D/A\right)$
Meso-Middle	$H_{\left(A_{1}\right)}\left(D/A\right)$	⋯	$H_{\left(A_{q}\right)}\left(D/A\right)$	⋯	$H_{\left(A_{n}\right)}\left(D/A\right)$	Macro-Top: H(D/A)

**Table 5:** Three bounds of double-granule conditional-entropies at meso-middle and macro-top.

U/IND(A)	$H_{\left(A_{p}\right)}\left(D/A\right)$	${\underline{H}}_{\left(A_{p}\right)}\left(D/A\right)$	${\bar{H}}_{\left(A_{p}\right)}\left(D/A\right)$	$H_{\left(A_{p}\right)}^{*}\left(D/A\right)$
$A_{1}$	$H_{\left(A_{1}\right)}\left(D/A\right)$	${\underline{H}}_{\left(A_{1}\right)}\left(D/A\right)$	${\bar{H}}_{\left(A_{1}\right)}\left(D/A\right)$	$H_{\left(A_{1}\right)}^{*}\left(D/A\right)$
⋮	⋮	⋮	⋮	⋮
$A_{p}$	$H_{\left(A_{p}\right)}\left(D/A\right)$	${\underline{H}}_{\left(A_{p}\right)}\left(D/A\right)$	${\bar{H}}_{\left(A_{p}\right)}\left(D/A\right)$	$H_{\left(A_{p}\right)}^{*}\left(D/A\right)$
⋮	⋮	⋮	⋮	⋮
$A_{n}$	$H_{\left(A_{n}\right)}\left(D/A\right)$	${\underline{H}}_{\left(A_{n}\right)}\left(D/A\right)$	${\bar{H}}_{\left(A_{n}\right)}\left(D/A\right)$	$H_{\left(A_{n}\right)}^{*}\left(D/A\right)$
Macro-Top	H(D/A)	$\underline{H}\left(D/A\right)$	$\bar{H}\left(D/A\right)$	$H^{*}\left(D/A\right)$

**Table 6:** A decision table.

*U*	$c_{1}$	$c_{2}$	$c_{3}$	$c_{4}$	$c_{5}$	$c_{6}$	$c_{7}$	$c_{8}$	$c_{9}$	$c_{10}$	$c_{11}$	*D*
$x_{1}$	2	2	4	4	4	3	4	4	4	2	4	1
$x_{2}$	2	2	4	4	2	2	4	4	4	2	3	1
$x_{3}$	3	4	3	4	2	4	2	4	4	2	2	0
$x_{4}$	2	4	2	3	2	4	2	4	2	2	4	0
$x_{5}$	4	4	2	4	2	4	3	4	4	4	3	0
$x_{6}$	2	4	2	4	2	2	2	4	4	4	4	0
$x_{7}$	2	2	4	4	2	2	2	3	4	4	2	0
$x_{8}$	2	2	4	4	2	2	2	4	4	3	4	1

**Table 7:** Information values of double-granule conditional-entropies in the example.

*U*	$A_{1}$	$A_{2}$	$A_{3}$	$A_{4}$	$A_{5}$	$A_{6}$	Meso-Middle
$A_{1}$	0	0.6887	0	0	0	0	0.6887
$A_{2}$	0.6887	0.9183	0.6887	0.6887	0.6887	0.6887	4.3619
$A_{3}$	0	0.6887	0	0	0	0	0.6887
$A_{4}$	0	0.6887	0	0	0	0	0.6887
$A_{5}$	0	0.6887	0	0	0	0	0.6887
$A_{6}$	0	0.6887	0	0	0	0	0.6887
Meso-Middle	0.6887	4.3619	0.6887	0.6887	0.6887	0.6887	Macro-Top: 7.8055

**Table 8:** Three bounds of double-granule conditional-entropies in the example.

*U*	$A_{1}$	$A_{2}$	$A_{3}$	$A_{4}$	$A_{5}$	$A_{6}$	Meso-Middle
$A_{1}$	[0,0] 0	[0.1722,0.9183] 0.8113	[0,0] 1	[0,0] 1	[0,0] 1	[0,0] 1	[0.1722,0.9183] 4.8113
$A_{2}$	[0.1722,0.9183] 0.8113	[0.3444,1.8366] 0.9183	[0.1722,0.9183] 1	[0.1722,0.9183] 1	[0.1722,0.9183] 1	[0.1722,0.9183] 1	[1.2053,6.4281] 5.7296
$A_{3}$	[0,0] 1	[0.1722,0.9183] 1	[0,0] 0	[0,0] 0	[0,0] 0	[0,0] 0	[0.1722,0.9183] 2.0000
$A_{4}$	[0,0] 1	[0.1722,0.9183] 1	[0,0] 0	[0,0] 0	[0,0] 0	[0,0] 0	[0.1722,0.9183] 2.0000
$A_{5}$	[0,0] 1	[0.1722,0.9183] 1	[0,0] 0	[0,0] 0	[0,0] 0	[0,0] 0	[0.1722,0.9183] 2.0000
$A_{6}$	[0,0] 1	[0.1722,0.9183] 1	[0,0] 0	[0,0] 0	[0,0] 0	[0,0] 0	[0.1722,0.9183] 2.0000
Meso-Middle	[0.1722,0.9183] 4.8113	[1.2053,6.4281] 5.7296	[0.1722,0.9183] 2.0000	[0.1722,0.9183] 2.0000	[0.1722,0.9183] 2.0000	[0.1722,0.9183] 2.0000	Macro-Top: [2.0662,11.0196] 18.5049

**Table 9:** Double-granule conditional-entropies based on an attribute-enlargement chain in the example.

Level	Measure	$CA_{1}$	$CA_{2}$	$CA_{3}$	$CA_{4}$	$CA_{5}$	$CA_{6}$	$CA_{7}$	$CA_{8}$	$CA_{9}$	$CA_{10}$	$CA_{11}$
Micro-Bottom	$H_{\left(CA_{k,1},CA_{k,1}\right)}\left(D/CA_{k,}\right)$ $H_{\left(CA_{k,1},CA_{k,2}\right)}\left(D/CA_{k}\right)$ $H_{\left(CA_{k,1},CA_{k,3}\right)}\left(D/CA_{k}\right)$ $H_{\left(CA_{k,2},CA_{k,2}\right)}\left(D/CA_{k}\right)$ $H_{\left(CA_{k,2},CA_{k,3}\right)}\left(D/CA_{k}\right)$	1.0000 0.8571 0.8571 0 0	0.8113 0.6490 0.5409 0 0	0.8113 0.6490 0.5409 0 0	0.8113 0.6490 0.6490 0 0	0 0.6887 0 0.9183 0.6887	0 0.6887 0 0.9183 0.6887	0 1 0 0 0	0 1 0 0 0	0 1 0 0 0	0 1 0 0 0	0 1 0 0 0
Meso-Middle	$H_{\left(CA_{k,1}\right)}\left(D/CA_{k}\right)$ $H_{\left(CA_{k,2}\right)}\left(D/CA_{k}\right)$ $H_{\left(CA_{k,3}\right)}\left(D/CA_{k}\right)$	2.7143 0.8571 0.8571	2.6502 0.6490 0.5409	2.6502 0.6490 0.5409	3.4074 0.6490 0.6490	0.6887 4.3619 0.6887	0.6887 4.3619 0.6887	0.6667 0.6667 0.6667	0 0 0	0 0 0	0 0 0	0 0 0
Macro-Top	$H(D/CA_{k})$ $\underline{H}\left(D/CA_{k}\right)$ $\bar{H}\left(D/CA_{k}\right)$ $H^{*}\left(D/CA_{k}\right)$	4.4286 2.2500 6.0000 4.9409	4.4891 1.6226 6.4902 6.6951	4.4891 1.6226 6.4902 6.6951	6.0035 2.0282 8.1128 8.5789	7.8055 2.0282 11.0196 18.5409	7.8055 2.0282 11.0196 18.5409	9.0000 1.7500 14.0000 28.0196	0 0 0 30	0 0 0 30	0 0 0 30	0 0 0 30

**Table 10:** Double-granule conditional-entropies regarding $CA_{2}=\left\{c_{1},c_{2}\right\}$ in the example.

*U*	$CA_{2,1}$	$CA_{2,2}$	$CA_{2,3}$	$CA_{2,4}$	Meso-Middle
$CA_{2,1}$	0.8113	0.6490	0.5409	0.6490	2.6502
$CA_{2,2}$	0.6490	0	0	0	0.6490
$CA_{2,3}$	0.5409	0	0	0	0.5409
$CA_{2,4}$	0.6490	0	0	0	0.6490
Meso-Middle	2.6502	0.6490	0.5409	0.6490	Macro-Top: 4.4891

**Table 11:** Three UCI data sets.

Label	Name	|U|	|C|	|U/IND(C)|	|D|	|U/IND(D)|
(1)	VOTING	435	16	342	1	2
(2)	SPECT	187	22	169	1	2
(3)	Tic-Tac-Toe	958	9	958	1	2

**Table 12:** Double-granule conditional-entropies in the VOTING data set.

$U/IND(CA_{1})$	$CA_{1,1}$	$CA_{1,2}$	$CA_{1,3}$	Meso-Middle	⋯	$U/IND(CA_{16})$	$CA_{16,1}$	⋯	$CA_{16,342}$	Meso-Middle
$CA_{1,1}$	0.9867	0.9782	0.8369	2.8018	⋯	$CA_{16,1}$	0	⋯	0	0
$CA_{1,2}$	0.9782	0.8113	0.6578	2.4473	⋯	⋮	⋮	⋱	⋮	⋮
$CA_{1,3}$	0.8369	0.6578	0.6479	2.1427	⋯	$CA_{16,342}$	0	⋯	0	0
Meso-Middle	2.8018	2.4473	2.1427	Macro-Top: 7.3918	⋯	Meso-Middle	0	⋯	0	Macro-Top: 0

**Table 13:** Three information bounds in the VOTING data set.

$U/IND(CA_{1})$	$CA_{1,1}$	$CA_{1,2}$	$CA_{1,3}$	Meso-Middle	⋯	$U/IND(CA_{16})$	$CA_{16,1}$	⋯	$CA_{16,342}$	Meso-Middle
$CA_{1,1}$	[1.0706, 1.9734] 0.9867	[0.5577, 1.7980] 0.9921	[0.8139, 1.6346] 0.9649	[2.4422, 5.4060] 2.9436	⋯	$CA_{16,1}$	[0,0] 0	⋯	[0,0] 0	[0,0] 221.3143
$CA_{1,2}$	[0.5577, 1.7980] 0.9921	[0.0448, 1.6226] 0.8113	[0.3009, 1.4592] 0.6596	[0.9034, 4.8798] 2.4630	⋮	⋮	⋮	⋱	⋮	⋮
$CA_{1,3}$	[0.8139, 1.6346] 0.9649	[0.3009, 1.4592] 0.6596	[0.5571, 1.2959] 0.6479	[1.6719, 4.3898] 2.2724	⋯	$CA_{16,342}$	[0,0] 0	⋯	[0,0] 0	[0,0] 221.3143
Meso-Middle	[2.4422, 5.4060] 2.9436	[0.9034, 4.8798] 2.4630	[1.6719, 4.3898] 2.2724	Macro-Top: [5.0174, 14.6755] 7.6790	⋯	Meso-Middle	[0,0] 221.3143	⋯	[0,0] 221.3143	Macro-Top: [0,0] 50132

**Table 14:** Double-granule conditional-entropies in the SPECT data set.

$U/IND(CA_{1})$	$CA_{1,1}$	$CA_{1,2}$	Meso-Middle	⋯	$U/IND(CA_{22})$	$CA_{22,1}$	⋯	$CA_{22,169}$	Meso-Middle
$CA_{1,1}$	0.2108	0.3815	0.5924	⋯	$CA_{22,1}$	0	⋯	0	1.5335
					⋮	⋮	⋱	⋮	⋮
$CA_{1,2}$	0.3815	0.5399	0.9215	⋯	$CA_{22,169}$	0	⋯	0	1.5335
Meso-Middle	0.5924	0.9215	Macro-Top: 1.5139	⋯	Meso-Middle	1.5335	⋯	1.5335	Macro-Top: 513.0879

**Table 15:** Three information bounds in the SPECT data set.

$U/IND(CA_{1})$	$CA_{1,1}$	$CA_{1,2}$	Meso-Middle	⋯	$U/IND(CA_{22})$	$CA_{22,1}$	⋯	$CA_{22,169}$	Meso-Middle
$CA_{1,2}$	[0.1015, 0.4217] 0.2109	[0.1908, 0.7508] 0.4030	[0.2922, 1.1725] 0.6138	⋯	$CA_{22,1}$	[0,0] 0	⋯	[0,0] 0	[0.0332, 1.9457] 8.8982
				⋮	⋮	⋮	⋱	⋮	⋮
$CA_{1,2}$	[0.1908, 0.7508] 0.4030	[0.2801, 1.0799] 0.5400	[0.4708, 1.8306] 0.9429	⋯	$CA_{22,169}$	[0,0] 0	⋯	[0,0] 0	[0.0332, 1.9457] 161.2140
Meso-Middle	[0.2922, 1.1725] 0.6138	[0.4708, 1.8306] 0.9429	Macro-Top: [0.7631, 14.6755] 1.5566	⋯	Meso-Middle	[0.0332, 1.9457] 8.8982	⋯	[0.0332, 1.9457] 161.2140	Macro-Top: [11.2085, 657.6332] 2867

**Table 16:** Double-granule conditional-entropies in the Tic-Tac-Toe data set.

$U/IND(CA_{1})$	$CA_{1,1}$	$CA_{1,2}$	$CA_{1,3}$	Meso-Middle	⋯	$U/IND(CA_{9})$	$CA_{9,1}$	⋯	$CA_{9,958}$	Meso-Middle
$CA_{1,1}$	0.8742	0.9248	0.8794	2.6784	⋯	$CA_{9,1}$	0	⋯	0	0
$CA_{1,2}$	0.9248	0.9881	0.9509	2.8638	⋯	⋮	⋮	⋱	⋮	⋮
$CA_{1,3}$	0.8794	0.9509	0.8901	2.7203	⋯	$CA_{9,958}$	0	⋯	0	0
Meso-Middle	2.6784	2.8638	2.7203	Macro-Top: 8.2625	⋯	Meso-Middle	0	⋯	0	Macro-Top: 0

**Table 17:** Three information bounds in the Tic-Tac-Toe data set.

$U/IND(CA_{1})$	$CA_{1,1}$	$CA_{1,2}$	$CA_{1,3}$	Meso-Middle	⋯	$U/IND(CA_{9})$	$CA_{9,1}$	⋯	$CA_{9,958}$	Meso-Middle
$CA_{1,1}$	[0.3814, 1.7483] 0.8741	[0.3635, 1.8622] 0.9404	[0.2859, 1.7642] 0.8796	[1.0308, 5.3748] 2.6940	⋯	$CA_{9,1}$	[0,0] 0	⋯	[0,0] 0	[0,0] 332
$CA_{1,2}$	[0.3635, 1.8622] 0.9404	[0.3455, 1.9762] 0.9881	[0.2680, 1.8781] 0.9628	[0.9770, 5.7165] 2.8913	⋮	⋮	⋮	⋱	⋮	⋮
$CA_{1,3}$	[0.2859, 1.7642] 0.8796	[0.2680, 1.8781] 0.9628	[0.1905, 1.7801] 0.8900	[0.7444, 5.4224] 2.7324	⋯	$CA_{9,958}$	[0,0] 0	⋯	[0,0] 0	[0,0] 626
Meso-Middle	[1.0308, 5.3748] 2.6940	[0.9770, 5.7165] 2.8913	[0.7444, 5.4224] 2.7342	Macro-Top: [2.7522, 16.5138] 8.3178	⋯	Meso-Middle	[0,0] 332	⋯	[0,0] 626	Macro-Top: [0,0] 415664
